# Aminobenzofuran-containing analogues of proximicins exhibit higher antiproliferative activity against human UG-87 glioblastoma cells compared to temozolomide†

**DOI:** 10.1039/d3ra00107e

**Published:** 2023-03-14

**Authors:** Nasrin Shokrzadeh Madieh, Sangeeta Tanna, Norah Ahmed Alqurayn, Alexandra Vaideanu, Andreas Schatzlein, Federico Brucoli

**Affiliations:** a Leicester School of Pharmacy, De Montfort University Leicester LE1 9BH UK federico.brucoli@dmu.ac.uk; b UCL School of Pharmacy, University College London 29/39 Brunswick Square London WC1N 1AX UK

## Abstract

A new series of proximicin analogues containing a benzofuran moiety as the replacement of the di-furan scaffold of the parent compound were synthesised and evaluated for their anti-proliferative activities against human glioblastoma cells U-87 MG. Proximicins A, B, and C are secondary metabolites produced by *Verrucosispora Fiedleri* MG-37, a Gram-positive actinomycete isolated from deep-sea sediment. Proximicins exhibit significant cytotoxic and apoptotic effects in a number of tumour cell lines, although further investigations on these natural products biological activity are hampered by the challenging synthesis of their constitutive di-furan unit. Therefore, the easily-synthesisable benzofuran ring was elected as a replacement of the di-furan platform, and a library of proximicin analogues was prepared in which different substituents were introduced at both the N-terminus and C-terminus of the benzofuran core unit. The novel compounds were tested against U-87 MG, as it was previously found that proximicins targeted this cancerous cell line, and the human healthy cell line WI-38. Temozolomide, the chemotherapeutic agent of choice for the treatment of glioblastoma, was used as a control. Analysis of growth inhibitory concentration values revealed that a number of furan-benzofuran-containing proximicin analogues, including 23(*16*) (IC_50 U-87 MG_ = 6.54 μg mL^−1^) exhibited higher antiproliferative activity against glioblastoma cells compared to both proximicins A–C and temozolomide (IC_50 U-87 MG_ = 29.19 μg mL^−1^) in U-87 MG.

## Abstract 2

A new series of proximicin analogues containing a benzofuran moiety as the replacement of the di-furan scaffold of the parent compound were synthesised and evaluated for their anti-proliferative activities against human glioblastoma cells U-87 MG.

## Graphical abstract

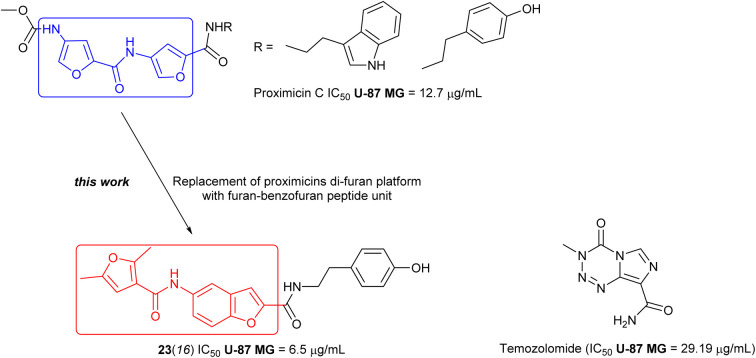

## Introduction

**Fig. 1:**
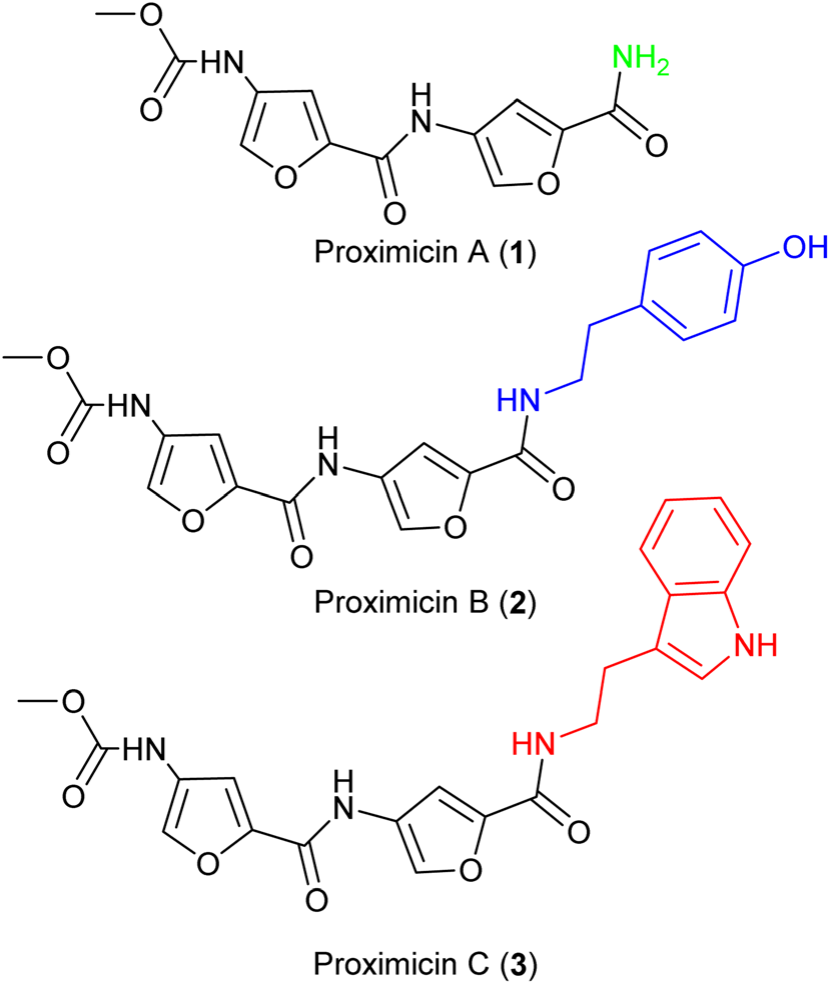
Structures of proximicins A (1), B (2) and C (3).

The molecular framework of bacterial secondary metabolites has long been utilised for the development of antitumour drugs.^1,2^ Proximicins A (1), B (2) and C (3) contain a characteristic 4-aminofuran-2-carboxylate structural motif, and were isolated from marine actinomycetes of the genus *Verrucosispora* (Fig. 1).^3,4^ Proximicins are heterocyclic-peptides endowed with significant biological properties. Proximicin B was found to induce apoptosis in both Hodgkin's lymphoma (L1236) and T-cell leukemia (Jurkat 16) cell lines, whereas proximicin C displayed significantly higher anti-proliferative activity against glioblastoma (U-87 MG) and breast carcinoma cells (MDA-MD-231) compared to proximicin B.^5,6^

Proximicin C (3) induced the up-regulation of cell-cycle regulatory proteins P53 and P21 in gastric adenocarcinoma cells.^5,6^ Further, the antimicrobial activities of proximicin B and C were evaluated *in vitro* against *Escherichia coli*, *Enterococcus faecalis* and drug-susceptible and -resistant *Staphylococcus aureus* strains. Proximicin B (2) displayed significant growth inhibition activity against the Gram-positive bacteria, including methicillin-resistant *S. aureus* EMRSA-15 and -16 strains at concentrations similar to those of selected tetracyclines.^6^

**Fig. 2:**
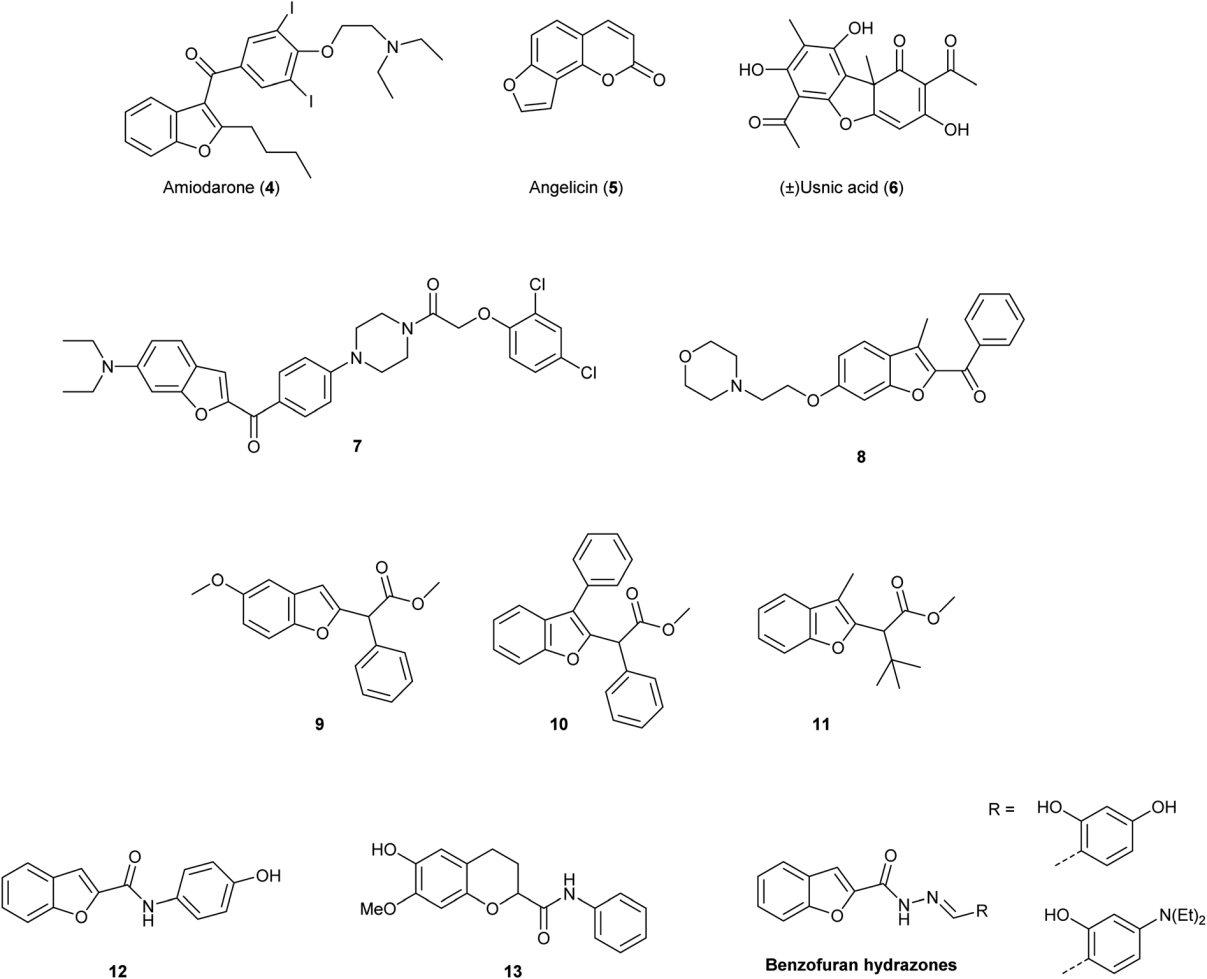
Molecular structures of benzofuran-containing approved drugs amiodarone (4), angelicin (5) and usnic acid (6), and bio-active synthetic compounds, *i.e.*, benzofuran-*N*-aryl piperazines (7), benzoyl-benzofurans (8), benzofuran-2-acetic acid esters (9–11) and *N*-phenyl-benzofuran-carboxamides (12).

Due to these relevant biological properties, we were interested in synthesising new series of proximicin analogues by replacing the di-furan scaffold with a benzofuran moiety. The synthesis of the di-furan peptide unit is challenging and low-yielding and prevents scale-up preparation of these marine natural products for further mechanistic studies or SAR investigations. As a result, we hypothesised that substitution of one of the proximicins furan rings with the readily-accessible benzofuran ring might furnish analogues with improved bio-activity compared to the parent compounds. Benzofuran is a privileged scaffold that can be found in the framework of marketed drugs and in a number of bio-active natural products and synthetic compounds (Fig. 2).^7–10^ For example, benzofuran is present in the structures of antiarrhythmic drug amiodarone (4) angelicin (5), which is a photosensitiser furocoumarin used for the treatment of psoriasis and other skin diseases, and usnic acid (6).^11^ Both enantiomers of usnic acid (6) are effective against a wide diversity of Gram-positive bacteria, especially the inhibition of the growth of multiresistant strain of *Staphylococcus aureus*, enterococci and mycobacteria.^12^

In addition, this important oxygen-containing bis-heterocycle ring can be found in structures of synthetic derivatives with significant cytotoxic activity. Benzofuran-*N*-aryl piperazine conjugates, such as 7, were found to exhibit remarkable cytotoxic activity against HeLa (IC_50_ = 0.03 μM), breast cancer MCF-7 (IC_50_ = 12.3 μM) and human gastric SGC-7901 (IC_50_ = 6.17 μM) cancer cell lines.^13^ Further, 2-benzoyl-3-methyl-6-(2-(morpholin-4-yl)ethoxy)benzofuran derivatives, *i.e*., 8, were active in anti-oestrogen receptor-dependent breast cancer cells MDA-MB-231 (IC_50_ = 8.36 μM).^14^ Moreover, simple benzofuran-2-acetic acid ester analogues 9, 10 and 11 were reported to inhibit breast cancer cells proliferation with pro-apoptotic activities being observed regardless of estrogen receptor dependency.^15^ Benzofuran derivatives 9, 10 and 11 induced apoptosis in both malignant T-cells and hepatocellular carcinoma cells, inhibited Aurora B kinase and VEGFR-2 activity, induced cell cycle arrest and reduced tubulin polymerization in lung and renal carcinoma cells.^15^*N*-Phenyl-benzofuran carboxamides (*i.e*., 12) and *N*-phenyl-2,3-dihydrobenzofuran-carboxamides (*i.e*., 13) exhibited inhibitory activity against a series of cancer cell lines (*e.g.*, ACHN, HCT15, MM231, NUGC-3, NCI-H23 and PC-3) with IC_50_ values ranging from 2.20–5.86 μM. *N*-Phenyl-benzofuran-carboxamide 12 also showed NF-κB inhibitory activity.^16^ Benzofuran-hydrazones were also found to exhibit growth inhibition at sub- and micro-molar levels against erythroleukemia K562 and Colo-38 melanoma human cells.^17^

Grade IV glioma or glioblastoma multiforme (GBM) is an aggressive type of cancer that affects the central nervous system.^18,19^ The estimated median survival is only 3–4 months, but this increases to about 15 months with treatment, which involves surgical resection, radiotherapy and temozolomide (TMZ) based pharmacological therapy.^20^ TMZ is the first-line therapeutic agent for GBM, but this alkylating agent is associated with significant side effects and the occurrence of drug resistance.^18,19^ There are currently very few alternatives to the standard therapy consisting of surgery followed by radiation and TMZ administration, and there is a growing need for chemical probes effective in treating high-grade glioblastomas and shortening chemotherapy treatments.

We have previously shown that proximicins are very effective in inhibiting the growth of U-87 MG glioblastoma cells *in vitro*, with proximicin C displaying an IC_50_ value of 12.7 μg mL^−1^.^6^ Here, capitalising on these findings, and in an effort to identify a suitable pharmacophore to replace the di-furan scaffold of proximicins, we have prepared a library of amino-benzofuran containing analogues that were tested using the MTT cell proliferation against U-87 MG glioblastoma cells and WI-38 human fibroblasts.

## Results and discussion

### Chemistry

**Scheme 1:**
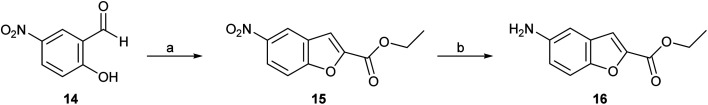
Reagents and conditions: (a) ethyl bromoacetate, dry K_2_CO_3_, dry DMF, 8 h, at reflux; (b) H_2_, 10% Pd/C, ethyl acetate, 3 h, rt.

**Scheme 2:**
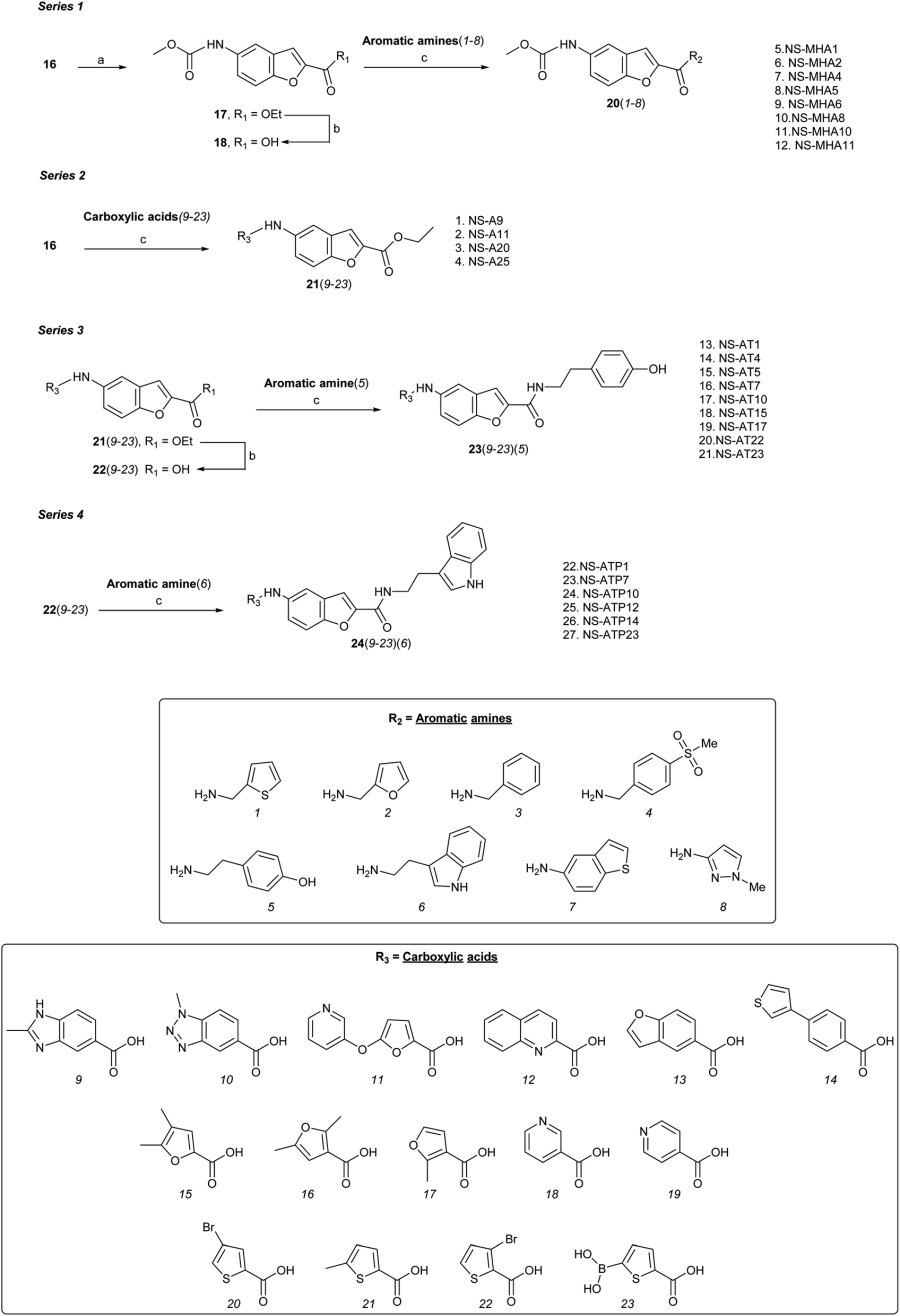
Reagents and conditions: (a) MeOCOCl, DIPEA, THF, 8 h, rt; (b) LiOH·H_2_O, H_2_O/THF (5 : 3), 3 h, rt; (c) EDCI–DMAP or EDCI–HOBt, DIPEA or HATU, DIPEA, dry CH_2_Cl_2_/DMF (2 : 1), 8–24 h, rt.

Four series (1–4) of novel derivatives were synthesised starting from the ethyl 5-amino benzofuran-2-carboxylate building block 16 (Schemes 1 and 2). The latter (16) was obtained in high yield (91%) after several reaction optimisation attempts. The optimised method involved initial *O*-alkylation of 5-nitrosalicylaldehyde (14) with a 3 molar excess of ethyl bromoacetate in the presence of 6 molar excess of anhydrous K_2_CO_3_, which was used as a basic dehydrating agent, under reflux for 8 hours. The resulting ethyl 5-nitrobenzofuran-2-carboxylate (15) was then converted to the amino-compound 16 using palladium (Pd/C) on activated coal as the catalyst under hydrogen stream (Scheme 1).

Series 1 analogues were synthesised by capping the N-terminus amino group of 16 with methyl chloroformate to give 17 (Scheme 2). After hydrolysis of the ester group, the free carboxylic acid 18 was coupled with aromatic/heteroaromatic amines (*1*–*8*) using the EDCI–DMAP (1.2 eq.) protocol in CH_2_Cl_2_ : DMF (2 : 1) to furnish 20(*1*–*8*). Peptide coupling of 16 with heteroaromatic carboxylic acids (*9*–*23*) using DIPEA and HATU gave series 2 compounds 21(*9*–*23*). Hydrolysis of ethyl ester function of 21(*9*–*23*) furnished acids 22(*9*–*23*), which were coupled with either tyramine to produce series 3 compounds 23(*9*–*23*), or tryptamine to give series 4 analogues 24(*9*–*23*). A total of 68 benzofuran-based proximicin-analogues were prepared and the structures of derivatives exhibiting most significant results are presented in Table S1.†

### Biological evaluation

The anticancer activity of the benzofuran derivatives was investigated using previously reported cellular methods.^6^ Prior to cytotoxicity evaluation, growth curve experiments were carried out at four different cell densities (500, 1000, 2000 and 4000 in 100 μL) to determine cell growth behaviour (Fig. S1†). The timeframe required for U-87 MG to enter the log phase was found to be three days at a cell density of 2000. This behaviour appeared to be influenced by the initial seeding density and this could be attributed to the cancerous nature of these cells.^21^ The WI-38 cells log phase entry was not influenced by initial cell density, and for 1000, 2000, or 4000, the time spent in the lag phase was 3 days, but the time spent in the log phase was 4, 5–6, and 7 days for 4000, 2000, 1000 respectively. Following a 6 day assay protocol, the cytotoxicity experiment could be conducted at both 1000 and 2000 cell densities for WI-38. Therefore, for both U-87 and WI-38, a 2000 cell density was found to be adequate for an estimation of the relative safety of the compounds.

The *in vitro* cytotoxicity of the proximicin analogues in U-87 cell lines was assessed using previously reported MTT protocols.^6,22,23^ Growth curves and cytotoxicity assay were conducted in triplicate. We carried out an initial screening in which cells were incubated with compounds for 24 h at a fixed concentration of 12 μg mL^−1^ to determine the percentage viability of U-87 MG and WI-38 cells (Table S1†). At this concentration (12 μg mL^−1^), temozolomide (TMZ) was found to reduce the growth of U-87 MG cells by around 41%, whereas other analogues such as 23(*16*) and 24(*15*) produced viability percentages of 33.62% and 39.43%, respectively. On the other hand, TMZ reduced the growth of WI-38 cells by almost 68% producing a viability % of ∼32.

**Fig. 3:**
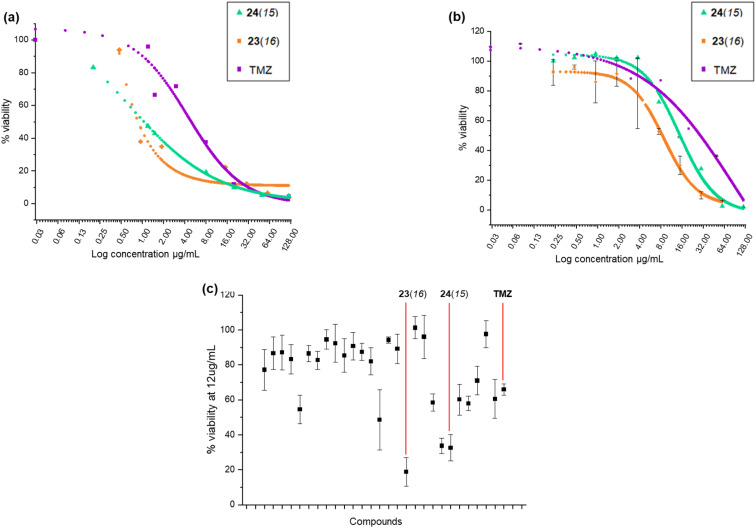
(a–c) Cytotoxicity profile. Log dose–response curve of 23(*16*) and 24(*15*) in (a) WI-38 and (b) U-87 MG cell lines; (c) screening of selected proximicins analogues and temozolomide (TMZ) at a single concentration of 12 μg mL^−1^ against U-87 MG. Replicates results were averaged and standard deviation (SD) was determined.

It was apparent from this initial screening that 24(*15*) 24(*16*), 24(*17*), 24(*21*) and 23(*16*) 23(*17*), which all contained N-terminal furan or thiophene ring and had either C-terminal tyrosine or tryptamine residues, inhibited U-87 MG cells proliferation at a higher rate compared to TMZ. The two most active proximicin derivatives 23(*16*) and 24(*15*) contained in their scaffolds N-terminal di-methyl furan rings (with different substitution patterns) and C-terminal tyramine [23(*16*)] or tryptamine [24(*15*)] residues, which are structural components of the proximicins (Fig. 3c). This indicates that these heterocyclic moieties are essential for the anti-proliferative activity of the compounds in U-87 MG cells. Interestingly, the substitution patterns of the N-terminal furan rings played a key role in modulating the growth inhibitory activity of the analogues. As can be noted, the presence of a single methyl group at the 2-position of N-terminal furan rings reduced the antiproliferative activity of compounds 23(*17*) and 24(*17*) in U-87 MG cells, whereas 2,5-dimethyl [23(*16*)] and 4,5-dimethyl [24(*15*)] substitution patterns conferred enhanced anti-proliferative properties to analogues 23(*16*) and 24(*15*). Further, inclusion of a N-terminal benzofuran residue yielded a derivative, 24(*13*), which exhibited reduced activity. As a result, 23(*16*) and 24(*15*) were further investigated and dose–response curves (Fig. 3a and b) were plotted to determine IC_50_ values. Derivative 23(*16*) was the most effective at reducing the growth of U-87 cells with an IC_50_ value of 6.54 μg mL^−1^ (15.67 μM), whereas 24(*15*) had an IC_50_ value of 15.02 μg mL^−1^ (34.9 μM). Temozolomide displayed an IC_50_ value of 29.19 μg mL^−1^ (150.34 μM) (Table 1). The compounds' toxicity was measured against non-cancerous WI-38 fibroblasts and it was found that temozolomide had an IC_50_ value of 4.94 μg mL^−1^ (25.4 μM), whereas 23(*16*) and 24(*15*) had IC_50_ values of 0.87 μg mL^−1^ (2.1 μM) and 1.05 μg mL^−1^ (2.4 μM), respectively.

**Table 1:** Comparison of percentage of growth inhibition and IC_50_ values for compounds 23(*16*), and 24(*15*) and TMZ

Compound ID	Percentage of inhibition at 12 μg mL^−1^		IC_50_ (μg mL^−1^)		Selectivity index IC_50 WI-38_/IC_50 U-87 MG_
	U-87 MG	WI-38	U-87 MG	WI-38	
23(*16*)	66.38%	24.4%	6.54	0.87	0.15
24(*15*)	60.57%	42.6%	15.02	1.05	0.07
TMZ	40.65%	68.07%	29.19	4.94	0.16

The selectivity index (S. I.) was calculated as the ratio between IC_50_ value for non-cancerous (WI-38) cells and IC_50_ value for cancerous (UG-87 MG) cells after treatments with TMZ, and analogues 23(*16*) and 24(*15*). Although data are not directly comparable as the compounds do not share the same mode of action, it can be noted that 23(*16*) had a S. I. of 0.15, and was >5-fold more active against glioblastoma cells compared to the first line glioblastoma drug TMZ, which had a S. I. of 0.16.

## Conclusions

A series of new proximicin analogues, in which the parent compound di-furan unit was replaced by a furan-benzofuran group, were synthesised in moderate to good yield and characterised by spectroscopic and spectrometry analysis. The synthesis of amino-benzofuran carboxylate was achieved in high yield after two-synthetic steps. It was found that this building block was an appropriate replacement for the amino-furan carboxylate, whose synthesis proved to be challenging. Mono- or dimethyl substituted furan ring attached *via* a peptide bond to the benzofuran unit of this chemotype furnished derivatives more active than the parent compounds (*e.g.*, proximicins). The novel proximicins analogues were tested for antiproliferative activity against U-87 MG cells and 23(*16*) inhibited the growth of this cancer cell line at concentration as low as 6.54 μg mL^−1^ (IC_50_), which was >5-fold lower than that of TMZ. Analogue 23(*16*) was 2-fold more active against U-87 MG cells compared to proximicin C.^6^ Further, 23(*16*) had a S. I. value comparable to that of TMZ. This is a promising result, especially considering the need to identify suitable therapeutic agents that can be used as an alternative to TMZ for the treatment of glioblastoma multiforme, which is an aggressive form of cancer with a five-year survival rate of only 10%. TMZ has a number of significant side effects and resistance to the alkylating drug often occurs due to DNA repair mechanisms. Finding new probes with a different mode of action compared to TMZ might solve issues related to resistance and lead to safer therapies against glioblastoma. The compounds are currently being investigated to elucidate their mechanism of action and will undergo a second round of medicinal chemistry work to decrease their cytotoxicity against non-cancerous cells.

## Author contributions

NSM, ST, NAA and AV conducted the experiments and collected data. AV supervised research activity and AS formulated research goals. FB conceptualised this study, designed and developed methods, and wrote the paper.

## Conflicts of interest

There are no conflicts to declare.

## Supplementary Material

RA-013-D3RA00107E-s001
