# Supplementary material for: Aminobenzofuran-containing analogues of proximicins exhibit higher antiproliferative activity against human UG-87 glioblastoma cells compared to temozolomide
Source: RSC Adv. 2023 Mar 14;13(12):8420–6. doi: 10.1039/d3ra00107e (PMC10012336; doi:10.1039/d3ra00107e)

Electronic Supplementary Material (ESI) for RSC Advances.
This journal is © The Royal Society of Chemistry 2023

Supporting Information

Aminobenzofuran-containing analogues of proximicins exhibit higher antiproliferative activity against human UG-87 glioblastoma cells compared to temozolomide

Table of Contents

Synthetic procedures page 2

MTT cytotoxicity assay page 14

Table S1. %viability of proximixicins analogues in U87-MG and WI-38 cells page 15

Growth curves for U87-MG and WI-38 cells page 18

Spectral data page 19

Experimental section

General information

Nuclear Magnetic Resonance (NMR) spectroscopy spectra were recorded on a JEOL JNM-ECZR 600 MHz (equipped with a ROYAL probe) and Bruker 400 Avance 400 MHz. Chemical shifts are reported in parts per million (ppm) with the solvent resonance as the internal standard and coupling constants (J) are quoted in Hertz (Hz). Splitting patterns were denoted as follows: s (singlet), br s (broad singlet), d (doublet), dd (doublet of doublets), t (triplet), q (quadruplet) and m (multiplet). LC-MS analysis was conducted on Thermo Fisher – Agilent 6100 series Quadrupole LC-MS system with a G4220A 1290 binary pump/DAD with a Kinetex C18 Phenomenex reversed-phase column (150 × 4.6 mm) and a flow rate of 0.5 mL min^{-1} and an eluent gradient of A (5-95%) over 15 min. Eluent A: CH_3CN/0.1% formic acid; Eluent B: H_2O/0.1 % formic acid. The total run time was 15 min with an injection volume of 20 µL. UHPLC-HRMS were acquired on an Agilent 1290 UHPLC coupled to an Agilent 6530 QTOF mass spectrometer used in the TOF mode. The total run time was 2.51 min and the injection volume was 5 µL. The mass spectrometer was operated in electrospray positive ion mode. Calibration of the TOF mass spectrometer was performed daily before analyses. HRMS reference masses: 121.0508 m/z and 922.0098 m/z. TLC was performed on Merk silica gel 60 F_{254} aluminium sheets. Column flash chromatography was performed on 40-63 µm particle size, 60 Å silica gel with ethyl acetate / hexane mixture as the eluent. All reagents were supplied by Sigma-Aldrich, Fischer Scientific, Merk and Alfa-Aesar in standard research grade. Human cancer cell line U-87 MG and human non-cancer cell line WI-38 were obtained from the American Type Culture Collection (ATCC) and cultured in 75 cm^3 flasks using Dulbecco's Modified Eagle Medium (DMEM) supplemented with 10% of Fetal Bovine Serum (FBS). DMEM was purchased from Sigma-Aldrich and FBS was purchased from Thermo Fisher.

Synthetic procedures

Ethyl 5- amino benzofuran-2-carboxylate (16). Benzofuran scaffold was synthesised according to previous methods[23] from 5-nitrosalicylaldehyde (**14**). In a round-bottom flask fitted with a condenser and charged with DMF were added **14** (100 mg, 0.59 mmol), ethyl bromoacetate (295 mg, 1.77 mmol) and K_2CO_3 (489 mg, 3.5 mmol). The reaction mixture was allowed to stir under reflux for 8 hours. After the reaction was completed as determined by TLC analysis, the mixture was poured into ice-cold water and extracted with DCM (3 × 2mL), washed with water (3 × 20mL), brine (3 × 20 mL) and dried over $MgSO_4$. The solvent was evaporated *in vacuo*, and the resulting crude solid was purified by flash chromatography (Hexane:EtOAc / 9:1) to obtain (**15**) 5-nitro ethylbenzofuran-2-carboxylate (LC-MS: m/z = 235.19 [M+]). For the subsequent hydrogenation reaction, **15** was dissolved in 30 mL ethyl acetate and evacuated and flushed with nitrogen before adding 10-mol% palladium on carbon (Pd/C).

The reaction mixture was left to react under hydrogen for 3 hours, filtered through a celite pad and evaporated to dryness to give **16** as light brown solid. The compound was characterised by LC-MS and ^{1}H-NMR spectroscopy. LC-MS: *m/z* (ES+) 206.19 [M+H]+. ^{1}H-NMR(400 MHZ, DMSO-D6) δ 7.50 (d, J = 1.Hz, 1H), 7.36 (dd, J = 1.04 Hz,1H), 6.81 (dd, J = 0.54, 1.46 Hz, 1H), 5.05 (s, J = 1.94 Hz, 1H), 4.32 (q, J = 2.17 Hz, 2H), 3.168 (d, J = 0.63 Hz, 2H), 1.31 (t, J = 3.27 Hz, 3H).

Ethyl 5-(methoxycarbonylamino)benzofuran-2-carboxylate (17). In a round-bottom flask, to a solution of **16** (100 mg, 0.487 mmol) in dry THF was added DIPEA (125 mg, 169,76 µL, 0.97 mmol) under a nitrogen atmosphere. After stirring at room temperature for 10 min, MeOCOCl (110 mg, 94.07 µL, 1.21 mmol, dissolved in 1 mL dry THF) was added dropwise and the reaction mixture was allowed to stir at 70 °C for 1.5 hours. After evaporation, the residue was dissolved in EtOAc (20 mL) and then washed with H_2O (3 × 20 mL), 10% HCl (3 × 20 mL), $NaHCO_3$ (3 × 20 mL) and brine (3 × 20 mL). The solvent was evaporated, and the crude solid was purified by flash chromatography (Hex:EtOAc / 7:3) to obtain 95.6 mg (96.20%) of **17.** LC-MS: *m/z* (ES+) 264.07 [M+H]+. ^{1}H-NMR (600 MHz, DMSO-D6) δ 9.76 (s, 1H), 7.95 (s, 1H), 7.74 (s, 1H), 7.64 (d, J = 9.0 Hz, 1H), 7.50 (d, J = 9.0 Hz, 1H), 4.35 (q, J = 7.1 Hz, 2H), 3.68 (s, 3H), 1.33 (t, J = 7.1 Hz, 3H).

5-(methoxycarbonylamino)benzofuran-2-carboxylic acid (18). To a solution of **17** (95.6 mg, 0.363 mmol) in THF (15 mL) was added dropwise LiOH•H_2O (114 mg, 2.72 mmol dissolved in 3 mL of H_2O). The reaction was allowed to react for 3 hours at room temperature. The solvent was evaporated, and the residue was treated with H_2O (5 mL). After acidification by 10% HCl to pH 3, the solution was extracted with EtOAc (3 × 20 mL), dried over $MgSO_4$ and concentrated in vacuo to obtain 68.6 mg (80.12%) of **18** as a white solid. LC-MS: *m/z* (ES+) 235.07 [M+]. ^{1}H-NMR (600 MHz, DMSO-D6) δ 9.70 (s, 1H), 7.88 (s, 1H), 7.58-7.56 (m, 2H), 7.43 (dd, J = 9.0, 2.1 Hz, 1H), 3.64 (s, 3H).

General procedure for amide coupling reactions

To a solution of the appropriate heterocyclic carboxylic acid (*9-23*, **Scheme 2**) in dry CH_2Cl_2:DMF (2:1) were added either EDCI-DMAP, or HATU, or EDCI-HOBt, DIPEA and left to stir for 15 min under nitrogen atmosphere, before adding amino benzofuran **16**. The reaction mixture was then allowed to react for 24h at room temperature. After consumption of the starting material, the solution was poured into ice-cold H_2O, extracted with EtOAc (3 × 20 mL), washed with H_2O (3 × 10 mL), 10% HCl (3 × 10 mL), 1M $NaHCO_3$ (3 × 10mL), and brine (3 × 10mL), and dried over $MgSO_4$. the solvent was evaporated, and residues purified by flash chromatography.

General procedure for hydrolysis

To a solution of ester in THF (5 mL) was added dropwise LiOH•H_2O dissolved in 3 mL of H_2O. The reaction mixture was allowed to stir for 1.5-3 h at room temperature. The solvent was evaporated under

reduced pressure and the residue was treated with 5 mL of H_2O. After acidification by 10% HCl to pH3, the solution was extracted with EtOAc (3 × 20mL), dried over $MgSO_4$ and concentrated in vacuo to obtain the desired carboxylic acid.

NS-A9. Ethyl5-[(2-methyl-1*H*-benzimidazole-5-carbonyl)amino]benzofuran-2-carboxylate 21(*9*). 2-methyl-1*H*-benzimidazole-5-carboxylic acid (*1*) (38.5 mg, 0.219 mmol) in anhydrous CH_2Cl_2:DMF (2:1) was treated with DIPEA (76.3 µL, 0.438 mmol), HATU (111 mg, 0.292 mmol), and amino benzofuran **16** (30 mg, 0.146 mmol) according to the general procedure. The final product was purified by column chromatography using EtOAc:Hex (6:4, R_f: 0.24) to afford **21(*9*) (**0.07mmol, 48.50%). ^{1}H-NMR (600 MHz, ACETONE-D6) δ 8.69 (s, 1H), 8.06 (d, J = 1.0 Hz, 1H), 8.04 (dd, J = 9.0, 2.1 Hz, 1H), 7.99 (dd, J = 9.1, 3.3 Hz, 1H), 7.91 (d, J = 8.6 Hz, 1H), 7.87-7.85 (m, 1H), 7.79-7.77 (m, 1H), 7.72-7.69 (m, 1H), 5.08 (d, J = 6.2 Hz, 1H), 4.84 (q, J = 7.1 Hz, 2H), 4.16 (s, 1H), 2.55 (s, 3H), 1.84-1.82 (m, 3H), LC-MS: *m/z* (ES+) 364.3 [M+H]+. HRMS calc. for $C_{20}H_{17}N_3O_4$ 363.1219, found 363.1727.

NS-A11. Ethyl 5-(pyridine-3-carbonylamino)benzofuran-2-carboxylate 21(*18*). Nicotinic acid (*18*) (26.9 mg, 0.219 mmol) in anhydrous CH_2Cl_2:DMF (2:1) was treated with DIPEA (76.3 µL, 0.438 mmol), HATU (111 mg, 0.292 mmol), and amino benzofuran **16** (30 mg, 0.146 mmol) according to the general procedure. The final product was purified by column chromatography using EtOAc:Hex (9:1, R_f : 0.25) to obtain **21**(*18*) (0.057 mmol, 39.66%). ^{1}H-NMR (600 MHz, CHLOROFORM-D) δ 9.40 (s, 1H), 8.69 (s, 1H), 8.42 (d, J = 7.6 Hz, 1H), 8.24 (s, 1H), 7.63-7.57 (m, 3H), 7.51 (s, 1H), 7.08 (s, 0H), 4.45 (q, J = 7.2 Hz, 2H), 1.44 (t, J = 7.2 Hz, 3H). ^{13}C-NMR (151 MHz, CHLOROFORM-D) δ 160.2, 158.2, 154.9, 143.4, 133.9, 128.8, 125.0, 120.2, 112.6, 109.4, 85.0, 29.6-29.7. LC-MS: *m/z* (ES+) 311.0 [M+H]+. HRMS calc. for $C_{17}H_{14}N_2O_4$ 310.3090, found 310.3041.

NS-A20. **Ethyl 5-(pyridine-4-carbonylamino)benzofuran-2-carboxylate 21**(*19*). Isonicotinic acid (*19*) (26.9 mg, 0.219 mmol) in anhydrous CH_2Cl_2:DMF (2:1) was treated with DIPEA (76.3 µL, 0.438 mmol), HATU (111 mg, 0.292 mmol), and amino benzofuran **16** (30 mg, 0.146 mmol), according to the general procedure. The final product was purified by column chromatography using EtOAc:Hex (9:1, R_f : 0.21) to obtain **21**(*19*) (0.02 mmol, 15%). ^{1}H-NMR (600 MHz, ACETONE-D6) δ 10.31 (s, 1H), 9.18 (d, J = 5.5 Hz, 2H), 8.81 (d, J = 6.2 Hz, 1H), 8.30 (dd, J = 4.3, 1.5 Hz, 2H), 8.23 (dd, J = 9.0, 2.4 Hz, 1H), 8.08 (s, 1H), 8.05 (d, J = 9.0 Hz, 1H), 4.81 (q, J = 7.1 Hz, 2H), 1.79 (t, J = 7.1 Hz, 3H). LC-MS: *m/z* (ES+) 311.2 [M+H]+. HRMS calc. for $C_{17}H_{14}N_2O_4$ 310.3090, found 310.30406.

NS-A25. Ethyl 5-[[5-(3-pyridyloxy)furan-2-carbonyl]amino]benzofuran-2-carboxylate 21(*11*). 5-(3-pyridinyloxy)-2-furoic acid (*11*) (29.9 mg, 0.146 mmol) in anhydrous CH_2Cl_2:DMF was treated with EDCI (33.58 mg, 0.174 mmol), HOBt (23.51 mg, 0.174 mmol), DMAP (21.25 mg, 0.174 mmol) and **16** (30 mg, 0.146 mmol). The final product was purified by column chromatography using EtOAc:Hex (2:8, R_f: 0.20) to afford **21**(*11*) (0.011 mmol, 8 %). ^{1}H-NMR (600 MHz, DMSO-D6) δ 9.79 (s, 1H),

8.14 (d, J = 2.8 Hz, 1H), 8.04 (dd, J = 4.6, 1.2 Hz, 1H), 7.79 (d, J = 2.1 Hz, 1H), 7.34 (d, J = 1.2 Hz, 1H), 7.31 (dd, J = 9.1, 2.2 Hz, 1H), 7.27-7.24 (m, 2H), 7.07 (q, J = 4.4 Hz, 1H), 6.95 (d, J = 3.8 Hz, 1H), 5.57 (d, J = 3.8 Hz, 1H), 3.93-3.90 (m, 2H), 0.90-0.88 (m, 3H). LC-MS: *m/z* (ES+) 392.1 [M+H]+. HRMS calc. for $C_{21}H_{16}N_2O_6$ 392.1008, found 392.1009.

NS-MHA1. Methyl *N*-[2-(benzothiophen-5-ylcarbamoyl)benzofuran-5-yl]carbamate 20(*7*). Carboxylic acid **18** (30 mg, 0.127 mmol) in anhydrous CH_2Cl_2:DMF was treated with EDCI (29.34 mg, 0.153 mmol), DMAP (18.69 mg, 0.153 mmol) and 1-benzothiophen-5-amine (*7*) (18.95 mg, 0.127 mmol) according to the general procedure. The final product was purified by column chromatography using EtOAc:Hex (2:8, R_f: 0.20) to afford **20**(*7*) (27.78%). ^{1}H-NMR (600 MHz, CHLOROFORM-D) δ 8.41 (s, 1H), 8.38 (d, J = 2.1 Hz, 1H), 7.87 (d, J = 8.6 Hz, 1H), 7.57 (d, J = 0.7 Hz, 1H), 7.54 (dd, J = 8.6, 2.1 Hz, 1H), 7.51 (s, 1H), 7.50 (t, J = 2.8 Hz, 2H), 7.39 (d, J = 8.6 Hz, 1H), 7.36 (d, J = 1.7 Hz, 1H), 5.12 (t, J = 6.4 Hz, 1H), 3.81 (s, 3H). LC-MS: *m/z* (ES+) 367.1 [M+H]+. HRMS calc. for $C_{19}H_{14}N_2O_4S$ 366.0674, found 367.0815 [M+H]+.

NS-MHA2. Methyl *N*-[2-[(1-methylpyrazol-3-yl)carbamoyl]benzofuran-5-yl]carbamate 20(*8*). Carboxylic acid **18** (30 mg, 0.127mmol) in anhydrous CH_2Cl_2:DMF was treated with EDCI (29.34 mg, 0.153 mmol), DMAP (18.69 mg, 0.153 mmol), and amine 1-methyl-1H-pyrazol-3-amine (*8*) (12.33 mg, 0.127 mmol), according to the general procedure. The final product was purified by column chromatography using EtOAc:Hex (2:8, R_f: 0.24) to afford **20**(*8*) (26.09%). ^{1}H-NMR (600 MHz, CHLOROFORM-D) δ 8.84 (s, 1H), 7.85-7.83 (m, 1H), 7.52 (d, J = 1.0 Hz, 1H), 7.44 (d, J = 9.0 Hz, 1H), 7.34 (d, J = 7.6 Hz, 1H), 7.30 (d, J = 2.4 Hz, 1H), 6.81 (d, J = 2.1 Hz, 1H), 6.71 (s, 1H), 3.85 (s, 3H), 3.80 (s, 3H). LC-MS: *m/z* (ES+) 315.1 [M+H]+. HRMS calc. for $C_{15}H_{14}N_4O_4$ 314.1015, found 315.1397 [M+H]+.

NS-MHA4. Methyl *N*-[2-(2-furylmethylcabamoyl)benzofuran-5-yl]carbamate 20(*2*). Carboxylic acid **18** (30 mg, 0.127 mmol) in anhydrous CH_2Cl_2:DMF was treated with EDCI (29.34 mg, 0.153mmol), DMAP (18.69 mg, 0.153 mmol), and 1-(furan-2-yl)methanamine (*2*) (12.33 mg, 0.127 mmol), according to the general procedure. The final product was purified by column chromatography using EtOAc:Hex (4.5:5.5, R_f: 0.20) to afford **20**(*2*) (38.71%). ^{1}H-NMR (600 MHz, CHLOROFORM-D) δ 7.78 (s, 1H), 7.44 (d, J = 1.0 Hz, 1H), 7.40-7.39 (m, 2H), 7.33 (d, J = 8.3 Hz, 1H), 6.90 (s, 1H), 6.75 (s, 1H), 6.36-6.32 (m, 2H), 4.66 (d, J = 5.9 Hz, 2H), 3.79 (s, 3H). LC-MS: *m/z* (ES+) 315.2 [M+H]+. HRMS calc. for $C_{16}H_{14}N_2O_5$ 314.2970, found 314.2927.

NS-MHA5. Methyl *N*-[2-(2-thienylmethylcarbamoyl)benzofuran-5-yl]carbamate 20(*1*). Carboxylic acid **18** (30 mg, 0.127mmol) in anhydrous CH_2Cl_2:DMF was treated with EDCI (29.34 mg, 0.153mmol), DMAP (18.69 mg, 0.153 mmol), and amine 1-(thiophen-2-yl)methanamine (*1*) (14.37 mg, 0.127 mmol), according to the general procedure. The final product was purified by column chromatography using EtOAc:Hex (4.5:5.5, R_f: 0.20) to afford **20**(*1*) (42.72%). ^{1}H-NMR (600 MHz,

CHLOROFORM-D) δ 7.79 (s, 1H), 7.46 (d, J = 0.5 Hz, 1H), 7.39 (d, J = 9.0 Hz, 1H), 7.32 (d, J = 7.9 Hz, 1H), 7.27 (d, J = 1.0 Hz, 1H), 7.08 (q, J = 1.5 Hz, 1H), 6.99 (dd, J = 5.3, 3.4 Hz, 1H), 6.91 (t, J = 5.0 Hz, 1H), 6.67 (s, 1H), 4.84 (dd, J = 5.9, 0.7 Hz, 2H), 3.79 (s, 3H). LC-MS: *m/z* (ES+) 331.1 [M+H]+. HRMS calc. for $C_{16}H_{14}N_2O_4S$, 330.0674, found 331.0799 [M+H]+.

NS-MHA6. Methyl *N*[2-(benzylcarbamoyl)benzofuran-5-yl]carbamate 20(*3*). Carboxylic acid **18** (30 mg, 0.127mmol) in anhydrous CH_2Cl_2:DMF was treated with EDCI (29.34 mg, 0.153mmol), DMAP (18.69 mg, 0.153 mmol), 1-phenylmethanamine (*3*) (13.60 mg, 0.127 mmol), according to the general procedure. The final product was purified by column chromatography using EtOAc:Hex (4.5:5.5, R_f: 0.21) to afford **20**(*3*) (29.80%). ^{1}H-NMR (600 MHz, CHLOROFORM-D) δ 7.79 (s, 1H), 7.46 (s, 1H), 7.38 (q, J = 3.2 Hz, 5H), 7.33-7.30 (m, 2H), 6.90-6.88 (m, 1H), 6.67 (s, 1H), 4.67 (d, J = 5.9 Hz, 2H), 3.79 (s, 3H). LC-MS: *m/z* (ES+) 325.1 [M+H]+. HRMS calc. for $C_{18}H_{16}N_2O_4$ 324.3360, 324.33064.

NS-MHA8. Methyl *N*-[2-[(4-methylsulfonylphenyl)methylcabamoyl]benzofuran-5-yl]carbamate 20(*4*). Carboxylic acid **18** (30 mg, 0.127mmol) in anhydrous CH_2Cl_2:DMF was treated with EDCI (29.34 mg, 0.153 mmol), DMAP (18.69 mg, 0.153 mmol), and 1-(4-methanesulfonylphenyl)methanamine (*4*) (23.52 mg, 0.127 mmol), according to the general procedure. The final product was purified by column chromatography using EtOAc:Hex (7:3, R_f: 0.20) to afford **20**(*4*) (79.14%). ^{1}H-NMR (600 MHz, DMSO-D6) δ 9.73 (s, 1H), 9.38 (t, J = 6.0 Hz, 1H), 7.92-7.88 (m, 4H), 7.58 (dd, J = 8.4, 5.3 Hz, 3H), 7.54 (s, 1H), 4.56 (s, 2H), 3.68 (s, 3H), 3.18 (s, 3H). LC-MS: *m/z* (ES+) 403.1 [M+H]+. HRMS calc. for $C_{19}H_{18}N_2O_6S$ 402.42100, found 402.42102.

NS-MHA10. Methyl *N*-[2-[2-(4-hydroxyphenyl)ethylcarbamoyl]benzofuran-5-yl]carbamate 20(*5*)**.** Carboxylic acid **18** (30 mg, 0.127 mmol) in anhydrous CH_2Cl_2:DMF was treated with EDCI (29.34 mg, 0.153mmol), DMAP (18.69 mg, 0.153 mmol), and 4-(2-aminoethyl)phenol (*5*) (17.42 mg, 0.127 mmol), according to the general procedure. The final product was purified by column chromatography using EtOAc:Hex (1:1, R_f: 0.20) to afford **20**(*5*) (97%). ^{1}H-NMR (600 MHz, ACETONE-D6) δ 9.17 (s, 1H), 8.42 (d, J = 15.5 Hz, 2H), 8.13 (d, J = 7.9 Hz, 1H), 7.99 (dd, J = 9.0, 2.1 Hz, 1H), 7.90-7.87 (m, 2H), 7.84 (d, J = 8.3 Hz, 1H), 7.68 (s, 1H), 7.56-7.53 (m, 1H), 7.48-7.46 (m, 1H), 4.22-4.18 (m, 2H), 4.16 (s, 3H), 3.56 (t, J = 7.6 Hz, 2H). LC-MS: *m/z* (ES+) 355.1 [M+H]+. HRMS calc. for $C_{19}H_{18}N_2O_5$ 354.36200, found 354.35662.

NS-MHA11. Methyl *N*-[2-(propylcarbamoyl)benzofuran-5-yl]carbamate 20(*6*). Carboxylic acid **18** (30 mg, 0.127mmol) in anhydrous CH_2Cl_2: DMF was treated with EDCI (29.34 mg, 0.153mmol), DMAP (18.69 mg, 0.153 mmol), and 2-(1-H-indol-3-yl)ethan-1-amine (*6*) (20.34 mg, 0.127 mmol) was added, according to the general procedure. The final product was purified by column chromatography using EtOAc: Hex (6:4 R_f: 0.21) to afford **20**(*6*) (41.34%). ^{1}H-NMR (600 MHz, DMSO-D6) δ 9.71 (s, 1H), 9.19 (s, 1H), 8.70 (t, J = 5.9 Hz, 1H), 7.92 (q, J = 3.1 Hz, 1H), 7.55 (d, J = 9.0 Hz, 1H), 7.52 (q, J

= 3.2 Hz, 1H), 7.46 (d, J = 0.7 Hz, 1H), 7.02 (dd, J = 6.5, 2.1 Hz, 2H), 6.87 (s, 1H), 6.78 (s, 1H), 6.68 (dd, J = 6.5, 2.1 Hz, 1H), 3.68 (s, 3H), 3.44-3.40 (m, 2H), 2.73 (t, J = 7.6 Hz, 2H). LC-MS: *m/z* (ES+) 378.1 [M+H]+. HRMS calc. for $C_{21}H_{19}N_3O_4$ 377.1375, found 378.1579 [M+H]+.

NS-AT1. 5-[(4,5-dimethylfuran-2-carbonyl)amino]-*N*-[2-(4-hydroxyphenyl)ethyl]benzofuran-2-carboxamide 23(*15*).

Carboxylic acid (*15*) 4,5 dimethyl-2-furoic acid (20.52 mg, 0.146 mmol) in CH_2Cl_2: DMF 2:1 was treated with EDCI (33.58 mg, 0.175 mmol), DMAP (21.37 mg, 0.175 mmol), and **16** (30 mg, 0.146 mmol) was added, according to the general procedure, and purified (EtOAc: Hex 3:7) to afford 32.44mg intermediate, yield **21**(*15*) (67.80%). The compound was hydrolysed to give 20.40 mg (0.068 mmol) carboxylic acid **22**(*15*) in 68.78% yield. The acid was judged pure by TLC analysis (20.40 mg, 0.068mmol) and coupled with tyramine (*5*) (9.32mg, 0.068 mmol) according to the general procedure of amide- coupling. The final product was purified by column chromatography using EtOAc: Hex 3:7 (R_f: 0.24) to give the final product **23**(*15*) 14.89mg in: 52.21% yield. ^{1}H-NMR (600 MHz, DMSO-D6) δ 10.01 (s, 1H), 9.13 (s, 1H), 8.68 (t, J = 5.9 Hz, 1H), 8.14 (d, J = 2.1 Hz, 1H), 7.67 (dd, J = 9.1, 2.2 Hz, 1H), 7.56 (d, J = 9.0 Hz, 1H), 7.46 (d, J = 0.7 Hz, 1H), 7.10 (s, 1H), 6.99 (dd, J = 6.5, 1.7 Hz, 2H), 6.64 (dd, J = 6.5, 2.1 Hz, 2H), 2.27 (s, 3H), 1.95 (s, 3H). LC-MS: *m/z* (ES+) 419.1[M+H]+. HRMS calc. for $C_{24}H_{22}N_2O_5$ 418.44900, found 418.44188.

NS-AT4. *N*-[2-(4-hydroxyphenyl)ethyl]-5-[[4-(3-thienyl)benzoyl]amino]benzofuran-2-carboxamid 23(*14*). 4-(3-thienyl) benzoic acid (*14*) (29.82 mg, 0.389 mmol) was dissolved in CH_2Cl_2: DMF 2:1 and treated with EDCI (89.48 mg, 0.466 mmol), DMAP (56.93 mg, 0.466 mmol), and **16** (80 mg, 0.389 mmol) according to the general procedure. The mixture was purified by column chromatography using (EtOAc: Hex 3:7) to afford 109.73 mg of intermediate **21**(*14*) (yield 71.91%). The compound was hydrolysed to give 57.58 mg of carboxylic acid **22**(*14*) in 56.53% yield. The carboxylic acid (57.58 mg, 0.158 mmol) was judged pure by TLC analysis and coupled with tyramine (*5*) (21.73 mg, 0.158 mmol) according to the general procedure of amide- coupling. The final product was purified by column chromatography using EtOAc: Hex 3:7 (R_f : 0.20) to give 5.40 mg of final product **23**(*14*) (yield: 7.1%). ^{1}H-NMR (600 MHz, ACETONE-D6) δ 8.11 (d, J = 10.0 Hz, 1H), 7.92-7.90 (m, 2H), 7.85 (q, J = 1.4 Hz, 1H), 7.78 (dt, J = 8.5, 2.0 Hz, 2H), 7.60-7.58 (m, 2H), 7.09 (dd, J = 6.7, 1.9 Hz, 2H), 7.07-7.06 (m, 1H), 7.03 (dd, J = 6.4, 1.9 Hz, 2H), 6.77-6.74 (m, 3H), 3.90 (s, 1H), 3.60-3.56 (m, 2H), 3.49-3.45 (m, 1H), 3.36-3.33 (m, 2H). LC-MS: *m/z* (ES+) 483 [M+H]+. HRMS calc. for $C_{24}H_{22}N_2O_4S$ 482.55400, found 482.55028.

NS-AT5. *N*-[2-[2-(4-hydroxyphenyl)ethylcarbamoyl]benzofuran-5-yl]-1-methyl-benzotriazole-5-carboxamide 23(*10*). 1-methyl-1H-1,2,3-benzotriazole-5-carboxylic acid (*10*) (68.91 mg, 0.389 mmol) in CH_2Cl_2: DMF 2:1 was treated with EDCI (89.48 mg, 0.466 mmol) DMAP (56.93 mg, 0.466 mmol), HOBt (62.96 mg, 0.466 mmol), and **16** (80 mg, 0.389 mmol) according to the general procedure. The mixture was purified by column chromatography using EtOAc: Hex 7:3 to afford 76.1 mg

intermediate **21**(*10*) in 53.67% yield. The compound was hydrolysed to give 29.76 mg carboxylic acid **22**(*10*) in 42.40% yield. The carboxylic acid (29.76 mg, 0.088 mmol) was judged pure by TLC and coupled with tyramine (*5*) (12.07 mg, 0.088 mmol) according to the general procedure of amide-coupling. The mixture was purified by column chromatography using EtOAc: Hex (9.5:0.5, R_f: 0.25) to give 3.56 mg of **23**(*10*) (yield: 8.6%). ^{1}H-NMR (600 MHz, ACETONE-D6) δ 8.49 (t, J = 1.2 Hz, 1H), 8.12 (s, 1H), 8.08 (d, J = 1.4 Hz, 1H), 8.07 (d, J = 1.4 Hz, 1H), 7.94 (s, 1H), 7.81 (dd, J = 8.6, 0.7 Hz, 1H), 7.11 (d, J = 8.3 Hz, 2H), 6.77 (dd, J = 6.5, 2.1 Hz, 2H), 4.37 (s, 3H), 3.64-3.60 (m, 2H), 2.86 (t, J = 7.4 Hz, 2H), 2.78 (s, 3H). LC-MS: *m/z* (ES+) 456.1 [M+H]+. HRMS calc. for $C_{25}H_{21}N_5O_4$ 455.15935, found 455.15823.

NS-AT7. *N*-[2-(4-hydroxyphenyl)ethyl]-5-[(5-methylthiophene-2-carbonyl)amino]benzofuran-2-carboxamide 23(*21*). 5-methyl-2- thiophene carboxylic acid (*21*) (20.75 mg, 0.146 mmol) in CH_2Cl_2: DMF 2:1 was treated with EDCI (33.58 mg, 0.175mmol), DMAP (21.37 mg, 0.175 mmol), and **16** (30 mg, 0.146 mmol) according to the general procedure. The mixture was purified by column chromatography using EtOAc: Hex (3:7) to afford 16.5 9mg of intermediate **21**(*21*) in 34.5% yield. The compound was hydrolysed to give 13.8 mg carboxylic acid **22**(*21*) in 91% yield. The carboxylic acid (13.81 mg, 0.046 mmol), which was judged pure by TLC, was coupled with tyramine (*5*) (6.31 mg, 0.046 mmol) according to the general procedure of amide coupling to give, after column chromatography purification (EtOAc: Hex / 3:7, R_f: 0.28), 7.08 mg of **23**(*21*) (yield: 36.73%). ^{1}H-NMR (600 MHz, DMSO-D6) δ 9.76 (s, 1H), 8.72 (s, 1H), 8.28 (t, J = 5.7 Hz, 1H), 7.70 (d, J = 2.1 Hz, 1H), 7.39 (d, J = 3.8 Hz, 1H), 7.23 (dd, J = 9.0, 2.1 Hz, 1H), 7.17 (d, J = 9.0 Hz, 1H), 7.06 (d, J = 0.7 Hz, 1H), 6.58 (d, J = 8.6 Hz, 2H), 6.48 (dd, J = 3.6, 1.2 Hz, 1H), 6.23 (dd, J = 6.5, 2.1 Hz, 2H), 3.00-2.96 (m, 3H), 2.94-2.93 (m, 1H), 2.29 (t, J = 7.6 Hz, 2H). LC-MS: *m/z* (ES+) 421.2 [M+H]+. HRMS calc. for $C_{23}H_{20}N_2O_4S$ 420.48300, found 420.4809.

NS-AT10. 5-[(2,5-dimethylfuran-3-carbonyl)amino]-*N*-[2-(4-hydroxyphenyl)ethyl]benzofuran-2-carboxamide 23(*16*). 2,5-dimethyl-3-furoic acid (*16*) (20.46 mg, 0.146 mmol) was dissolved in CH_2Cl_2: DMF 2:1 and treated with EDCI (33.58 mg, 0.175 mmol), DMAP (21.37 mg, 0.175 mmol), and **16** (30 mg, 0.146 mmol) according to the general procedure. The mixture was purified by column chromatography using (EtOAc: Hex 3:7) to afford 21.64 mg of intermediate **21**(*16*) in 45.28% yield. The compound was hydrolysed to give 15.63 mg carboxylic acid **22**(*16*) in 79% yield. The carboxylic acid (15.63 mg, 0.052 mmol), which was judged pure by TLC, was coupled with tyramine (*5*) (7.13 mg, 0.052 mmol) according to the general procedure of amide-coupling to give, after column chromatography purification (EtOAc: Hex 6:4 R_f : 0.26), 11.43 mg of final product **23**(*16*) (yield: 52.57%). ^{1}H-NMR (600 MHz, DMSO-D6) δ 9.65 (s, 1H), 9.13 (s, 1H), 8.68 (t, J = 5.7 Hz, 1H), 8.15 (d, J = 2.1 Hz, 1H), 7.62 (dd, J = 9.0, 2.4 Hz, 1H), 7.55 (d, J = 9.0 Hz, 1H), 7.46 (d, J = 0.7 Hz, 1H), 6.99 (dd, J = 6.5, 2.1 Hz, 1H), 6.64 (dd, J = 6.5, 2.1 Hz, 3H), 3.41-3.37 (m, 2H), 3.34 (d, J = 1.0 Hz,

0H), 2.70 (t, J = 7.6 Hz, 2H), 2.47 (s, 3H), 2.23 (s, 3H). LC-MS: *m/z* (ES+) 419.1 [M+H]+. HRMS calc. for $C_{24}H_{22}N_2O_5$ 418.44900, found 418.44188.

NS-AT15. [5-[[2-[2-(4-hydroxyphenyl)ethylcarbamoyl]benzofuran-5-yl]carbamoyl]-2-thienyl]boronic acid 23(*23*). 5-(dihydroxyboryl)-2-thiophene carboxylic acid (*23*) (66.89 mg, 0.389 mmol) in CH_2Cl_2: DMF 2:1 was treated with EDCI (89.48 mg, 0.466 mmol), DMAP (56.93 mg, 0.466 mmol), and **16** (80 mg, 0.389 mmol) according to the general procedure. The mixture was purified by column chromatography using EtOAc: Hex (7:3) to afford 66.64 mg of intermediate **21**(*23*) in 47.7% yield. The compound was hydrolysed to give 45.27 mg carboxylic acid **22**(*23*) in 73.69% yield. The carboxylic acid (45.27 mg, 0.136 mmol), which was judged pure by TLC, was coupled with tyramine (*5*) (18.65 mg, 0.136 mmol) according to the general procedure of amide-coupling to give, after column chromatography purification (EtOAc: Hex 7:3 R_f: 0.25), 7.64 mg of final product **23**(*23*) (yield: 12.41%). ^{1}H-NMR (600 MHz, DMSO-D6) δ 9.88 (s, 1H), 8.72 (s, 1H), 8.29 (t, J = 5.7 Hz, 1H), 7.72 (d, J = 2.4 Hz, 1H), 7.58 (dd, J = 3.8, 1.0 Hz, 1H), 7.41 (dd, J = 4.8, 1.0 Hz, 1H), 7.25 (dd, J = 9.0, 2.1 Hz, 1H), 7.18 (d, J = 9.0 Hz, 1H), 7.07 (d, J = 1.0 Hz, 1H), 6.79 (dd, J = 4.7, 3.9 Hz, 1H), 6.58 (dd, J = 6.5, 2.1 Hz, 1H), 6.23 (dd, J = 6.4, 1.9 Hz, 1H), 2.98 (t, J = 4.3 Hz, 2H), 2.29 (t, J = 7.6 Hz, 2H). LC-MS: *m/z* (ES+) 451.1 M+H]+. HRMS calc. for $C_{22}H_{19}BN_2O_6S$ 450.27200, found 450.27206.

NS-AT17. 5-[(3-bromothiophene-2-carbonyl)amino]-*N*-[2-(4-hydroxyphenyl)ethyl]benzofuran-2-carboxamide 23(*22*). 3-bromothiophene-2-carboxylic acid (*22*) (80.68 mg, 0.389 mmol) in CH_2Cl_2: DMF 2:1 was treated with EDCI (89.48 mg, 0.466 mmol), DMAP (56.93 mg, 0.466 mmol), and **16** (80 mg, 0.389 mmol) according to the general procedure. The mixture was purified by column chromatography using EtOAc: Hex (2:8) to afford 55.88 mg of intermediate **21**(*22*) in 36.4% yield. The compound was hydrolysed to give 43.30 mg carboxylic acid **22**(*22*) in 83.43% yield. The carboxylic acid (43.30 mg, 0.118 mmol), which was judged pure by TLC, was coupled with tyramine (*5*) (16.18 mg, 0.118 mmol) according to the general procedure of amide-coupling to give, after column chromatography purification (EtOAc:Hex 6:4, R_f: 0.25) 4.56 mg of final product **23**(*22*) (yield: 7.95%). ^{1}H-NMR (600 MHz, CHLOROFORM-D) δ 8.96 (s, 1H), 8.26 (d, J = 2.4 Hz, 1H), 7.70 (d, J = 1.0 Hz, 1H), 7.61 (d, J = 8.6 Hz, 1H), 7.54-7.52 (m, 2H), 7.18 (dd, J = 6.5, 2.1 Hz, 2H), 7.11 (d, J = 5.2 Hz, 1H), 7.03 (d, J = 8.3 Hz, 2H), 6.75 (dd, J = 6.5, 2.1 Hz, 2H), 4.78 (s, 1H), 4.53 (d, J = 36.8 Hz, 1H), 3.35 (dd, J = 43.6, 6.0 Hz, 3H), 2.82 (t, J = 6.7 Hz, 2H), 2.70 (t, J = 7.1 Hz, 2H). LC-MS: *m/z* (ES+) 486.9 [M+H]+. HRMS calc. for $C_{22}H_{17}BrN_2O_4S$ 485.0092, found 485.0168.

NS-AT22.5-[(4-bromothiophene-2-carbonyl)amino]-*N*-[2-(4-hydroxyphenyl)ethyl]benzofuran-2-carboxamide 23(*20*). 4-bromothiophene-2-carboxylic acid (*20*) (30.22 mg, 0.146 mmol) in CH_2Cl_2: DMF 2:1 was treated with EDCI (33.58 mg, 0.175 mmol), DMAP (21.37 mg, 0.175 mmol), HOBt (23.64 mg, 0.175 mmol), and **16** (30 mg, 0.146 mmol) according to the general procedure. The mixture was purified by column chromatography using EtOAc:Hex (2:8) to afford 36.0 mg of intermediate

21(*20*) in 62.6% yield. The compound was hydrolysed to give 19.98 mg of carboxylic acid **22**(*20*) in 59.7% yield. The carboxylic acid (19.98 mg, 0.054 mmol), which was judged pure by TLC, was coupled with tyramine (*5*) (7.40 mg, 0.052 mmol) according to the general procedure of amide coupling to give, after column chromatography purification (EtOAc: Hex 6:4, R_f: 0.33) 19.77 mg of final product **23**(*20*) (yield: 74.66%). ^{1}H-NMR (600 MHz, ACETONE-D6) δ 9.70 (s, 1H), 8.24 (s, 1H), 8.17 (s, 1H), 7.92 (d, J = 1.4 Hz, 1H), 7.83 (d, J = 1.4 Hz, 1H), 7.76 (dd, J = 9.0, 2.1 Hz, 1H), 7.52 (d, J = 9.0 Hz, 2H), 7.46 (s, 1H), 7.11 (d, J = 8.3 Hz, 2H), 6.77 (dd, J = 6.2, 2.1 Hz, 1H), 5.18-5.16 (m, 2H), 3.64 (s, 1H), 3.63-3.60 (m, 2H), 3.20-3.20 (1H). LC-MS: *m/z* (ES+) 486.5 [M+H]+. HRMS calc. for $C_{22}H_{17}BrN_2O_4S$ 485.35200, found 485.35038.

NS-AT23. *N*-[2-(4-hydroxyphenyl)ethyl]-5-[(2-methylfuran-3-carbonyl)amino]benzofuran-2-carboxamide 23(*17*). 2-methyl-3-furoic acid (*17*) (18.41mg, 0.146mmol) in CH_2Cl_2: DMF 2:1 was treated with EDCI (33.58 mg, 0.175 mmol), DMAP (21.37 mg, 0.175 mmol), and **16** (30 mg, 0.146 mmol) according to the general procedure. The mixture was purified by column chromatography using EtOAc:Hex (2:8) to afford 31.1 mg of intermediate **21**(*17*) in 67.9%. yield. The compound was hydrolysed to give 21.5 mg carboxylic acid **22**(*17*) in 75.9% yield. The carboxylic acid (21. 5 mg, 0.075 mmol), which was judged pure by TLC, was coupled with tyramine (*5*) (10.28 mg, 0.075 mmol) according to the general procedure of amide coupling to give, after column chromatography purification (EtOAc:Hex 7:3, R_f : 0.28) 23.4 mg of final product **23**(*17*) (yield: 76.6%). ^{1}H-NMR (600 MHz, ACETONE-D6) δ 9.15 (s, 1H), 8.24 (s, 1H), 7.90 (t, J = 5.0 Hz, 1H), 7.75-7.70 (m, 1H), 7.48-7.46 (m, 2H), 7.44-7.43 (m, 1H), 7.11 (d, J = 8.6 Hz, 2H), 6.96 (d, J = 2.1 Hz, 1H), 6.77 (dt, J = 9.1, 2.4 Hz, 2H), 3.64-3.60 (m, 3H), 3.20 (s, 1H), 2.86 (d, J = 7.6 Hz, 3H), 2.61 (s, 3H). LC-MS: *m/z* (ES+) 405.1 [M+H]+. HRMS calc. for $C_{23}H_{20}N_2O_5$ 404.4220, found 404.4153.

NS-ATP1. 5-[(4,5-dimethylfuran-2-carbonyl)amino]-*N*-[2-(1H-indol-3-yl)ethyl]benzofuran-2-carboxamide 24(*15*). 4,5 dimethyl-2-furoic acid (*15*) (20.52 mg, 0.146 mmol) in CH_2Cl_2: DMF 2:1 was treated with EDCI (33.58 mg, 0.175 mmol) DMAP (21.37 mg, 0.175 mmol), and **16** (30 mg, 0.146 mmol), according to the general procedure. The mixture was purified by column chromatography using EtOAc:Hex (3:7) to afford 32.4 mg of intermediate **21**(*15*) in 67.8% yield. The compound was hydrolysed to give 20.4 mg of carboxylic acid **22**(*16*) in 68.7% yield. The resulting carboxylic acid (20.4 mg, 0.068 mmol) was coupled with tryptamine (*6*) (10.89 mg, 0.068 mmol) according to the general procedure of amide-coupling to give, after column chromatography purification (EtOAc:Hex 3:7, R_f: 0.26), 8.6 mg of final product **24**(*15*) (yield: 28.8%). ^{1}H-NMR (600 MHz, DMSO-D6) δ 10.77 (s, 1H), 10.01 (s, 1H), 8.77 (t, J = 5.9 Hz, 1H), 8.15 (d, J = 2.1 Hz, 1H), 7.67 (dd, J = 9.0, 2.4 Hz, 1H), 7.57-7.55 (m, 1H), 7.48-7.47 (m, 1H), 7.30 (d, J = 8.3 Hz, 1H), 7.15 (d, J = 2.1 Hz, 1H), 7.10 (s, 1H), 7.04-7.01 (m, 1H), 6.95-6.93 (m, 1H), 3.54-3.51 (m, 2H), 2.93 (t, J = 7.6 Hz, 2H), 2.27 (s, 2H), 1.95 (d, J = 2.1 Hz, 3H). LC-MS: *m/z* (ES+) 442.1 [M+H]+. HRMS calc. for $C_{26}H_{23}N_3O_4$ 441.1688, found 441.1834.

NS-ATP7. N-[2-(1H-indol-3-yl)ethyl]-5-[(5-methylthiophene-2-carbonyl)amino]benzofuran-2-carboxamide 24(*21*). 5-methyl-2- thiophene carboxylic acid (*21*) (20.7 mg, 0.146 mmol) in CH_2Cl_2:DMF 2:1 was treated with EDCI (33.6 mg, 0.175 mmol) DMAP (21.4 mg, 0.175 mmol), and **16** (30.1 mg, 0.146 mmol), according to the general procedure. The mixture was purified by column chromatography using EtOAc:Hex (3:7) to afford 16.6 mg of intermediate **21**(*21*) in 34.5% yield. The compound was hydrolysed to give 13.8 mg of carboxylic acid **22**(*21*) in 91% yield. The resulting carboxylic acid (13.8 mg, 0.046 mmol) was coupled with tryptamine (*6*) (7.36 mg, 0.046 mmol) according to the general procedure of amide- coupling to give, after column chromatography purification (EtOAc:Hex 3:7 R_f: 0.29), 4.7 mg of final product **24**(*21*) (yield: 23.25%). ^{1}H-NMR (600 MHz, DMSO-D6) δ 10.36 (s, 1H), 9.76 (s, 1H), 8.37 (t, J = 5.9 Hz, 1H), 7.71 (d, J = 2.1 Hz, 1H), 7.39 (d, J = 3.8 Hz, 1H), 7.24 (dd, J = 9.0, 2.1 Hz, 1H), 7.18-7.14 (m, 2H), 7.08 (d, J = 0.7 Hz, 1H), 6.89 (d, J = 8.3 Hz, 1H), 6.75 (d, J = 2.1 Hz, 1H), 6.62 (td, J = 7.6, 1.0 Hz, 1H), 6.53 (td, J = 7.4, 0.8 Hz, 1H), 6.48 (dd, J = 3.6, 1.2 Hz, 1H), 3.11 (dd, J = 14.3, 6.7 Hz, 2H), 2.52 (t, J = 7.6 Hz, 2H), 2.06 (s, 3H). LC-MS: *m/z* (ES+) 444.1 [M+H]+. HRMS calc. for $C_{25}H_{21}N_3O_3S$ 443.52100, found 443.51754.

NS-ATP10. **5-[(2,5-dimethylfuran-3-carbonyl)amino]-*N*-[2-(1H-indol-3-yl)ethyl]benzofuran-2-carboxamide 24**(*16*). 2,5-dimethyl-3-furoic acid (*16*) (20.5 mg, 0.146 mmol) in CH_2Cl_2:DMF 2:1 was treated with EDCI (33.6 mg, 0.175 mmol) DMAP (21.4 mg, 0.175 mmol), and **16** (30 mg, 0.146 mmol), according to the general procedure. The mixture was purified by column chromatography using EtOAc:Hex (3:7) to afford 21.6 mg intermediate **21**(*16*) in 45.3% yield. The compound was hydrolysed to give 15.6 mg of carboxylic acid **22**(*16*) in 79% yield. The resulting carboxylic acid (15.6 mg, 0.052 mmol) was coupled with tryptamine (*6*) (8.33 mg, 0.052 mmol) according to the general procedure of amide-coupling to give, after column chromatography purification (EtOAc:Hex 6:4, R_f: 0.24), 5.8 mg of final product **24**(*16*) (yield: 25.3%). ^{1}H-NMR (600 MHz, ACETONE-D6) δ 10.69 (s, 1H), 8.62 (d, J = 8.6 Hz, 1H), 8.48 (d, J = 2.1 Hz, 1H), 8.37 (d, J = 8.6 Hz, 1H), 8.21 (d, J = 8.3 Hz, 1H), 8.11 (d, J = 7.6 Hz, 1H), 7.93-7.90 (m, 1H), 7.77-7.75 (m, 1H), 7.69 (t, J = 4.0 Hz, 2H), 7.58 (dd, J = 13.3, 8.4 Hz, 2H), 7.51 (s, 1H), 7.38 (q, J = 8.0 Hz, 2H), 7.25 (d, J = 2.1 Hz, 1H), 7.15 (d, J = 1.7 Hz, 1H), 7.12-7.07 (m, 1H), 7.04-6.99 (m, 2H), 3.77 (td, J = 7.4, 6.2 Hz, 2H), 3.47-3.51 (1H), 3.13 (t, J = 7.6 Hz, 2H). LC-MS: m/z (ES+) 442.1 [M+H]+. HRMS calc. for $C_{26}H_{23}N_3O_4$ 441.48700, found 441.47852.

NS-ATP12. *N*-[2-[2-(1*H*-indol-3-yl)ethylcarbamoyl]benzofuran-5-yl]quinoline-2-carboxamide 24(*12*). 2-quinoline carboxylic acid (*12*) (67.4 mg, 0.389 mmol) in CH_2Cl_2:DMF 2:1 was treated with EDCI (89.5 mg, 0.466 mmol) DMAP(56.9 mg, 0.466 mmol), HOBt (62.9 mg, 0.466 mmol), and **16** (80 mg, 0.389 mmol), according to the general procedure. The mixture was purified by column chromatography using EtOAc: Hex (3:7) to afford 134.1 mg of intermediate **21**(*12*) in 95.7% yield. The compound was hydrolysed to give 70.3 mg of carboxylic acid **22**(*12*) in 56.8% yield. The resulting carboxylic acid (70.3 mg, 0.211 mmol) was coupled with tryptamine (*6*) (33.9 mg, 0.211 mmol) according to the general procedure of amide-coupling to give, after column chromatography

purification (EtOAc:Hex 6:4, R_f: 0.33), 10.1 mg of final product **24**(*12*) (yield: 10%). ^{1}H-NMR (600 MHz, ACETONE-D6) δ 10.69 (s, 1H), 8.62 (d, J = 8.6 Hz, 1H), 8.48 (d, J = 2.1 Hz, 1H), 8.37 (d, J = 8.6 Hz, 1H), 8.21 (d, J = 8.3 Hz, 1H), 8.11 (d, J = 7.6 Hz, 1H), 7.93-7.90 (m, 1H), 7.77-7.75 (m, 1H), 7.69 (t, J = 4.0 Hz, 2H), 7.58 (dd, J = 13.3, 8.4 Hz, 2H), 7.51 (s, 1H), 7.38 (q, J = 8.0 Hz, 2H), 7.25 (d, J = 2.1 Hz, 1H), 7.15 (d, J = 1.7 Hz, 1H), 7.12-7.07 (m, 1H), 7.04-6.99 (m, 2H), 3.77 (td, J = 7.4, 6.2 Hz, 2H), 3.47-3.51 (1H), 3.13 (t, J = 7.6 Hz, 2H) LC-MS: *m/z* (ES+) 475.3 [M+H]+. HRMS calc. for $C_{29}H_{22}N_4O_3$ 474.1691, found 474.1881.

NS-ATP14 5-(benzofuran-5-carbonylamino)-*N*-[2-(1*H*-indol-3-yl)ethyl]benzofuran-2-carboxamide 24(*13*). Benzofuran-5-carboxylic acid (*13*) (63.1 mg, 0.389 mmol) in CH_2Cl_2:DMF 2:1 was treated with EDCI (89.5 mg, 0.466 mmol), DMAP (56.9 mg, 0.466 mmol), HOBt (62.9 mg, 0.466 mmol), and **16** (80 mg, 0.389 mmol) according to the general procedure. The mixture was purified by column chromatography using EtOAc:Hex (4:6) to afford 52.6 mg of intermediate **21**(*13*) in 38.7% yield. The compound was hydrolysed to give 31.7 mg carboxylic acid **22**(*12*) in 65.5% yield. The resulting carboxylic acid (31.7 mg, 0.098 mmol) was coupled with tryptamine (*6*) (15.7 mg, 0.098 mmol) according to the general procedure of amide-coupling to give after column chromatography purification (EtOAc:Hex 6:4, R_f: 0.24), 3.7 mg of final product **24**(*13*) (yield: 8%). ^{1}H-NMR (600 MHz, ACETONE-D6) δ 10.02 (s, 1H), 9.68 (s, 1H), 8.35 (q, J = 1.8 Hz, 2H), 8.02 (dd, J = 8.6, 1.7 Hz, 1H), 7.96 (d, J = 2.4 Hz, 1H), 7.83 (dd, J = 9.0, 2.1 Hz, 1H), 7.67 (q, J = 8.4 Hz, 2H), 7.51 (d, J = 9.0 Hz, 1H), 7.48 (d, J = 1.0 Hz, 1H), 7.40-7.38 (m, 1H), 7.24 (d, J = 2.1 Hz, 1H), 7.12-7.09 (m, 1H), 7.04-7.01 (m, 3H), 3.76 (td, J = 7.5, 6.1 Hz, 2H), 3.12 (t, J = 7.6 Hz, 2H). LC-MS: *m/z* (ES+) 464.1 [M+H]+. HRMS calc. for $C_{28}H_{21}N_3O_4$ 463.4900, found 463.48404.

NS-ATP23. *N*-[2-(1*H*-indol-3-yl)ethyl]-5-[(2-methylfuran-3-carbonyl)amino]benzofuran-2-carboxamide 24(*17*). 2-methyl-3-furoic acid (*17*) (18.4 mg, 0.146 mmol) in CH_2Cl_2:DMF 2:1 was treated with EDCI (33.6 mg, 0.175 mmol), DMAP (21.4 mg, 0.175 mmol), HOBt (23.6 mg, 0.175mmol), and **16** (30.1 mg, 0.146 mmol) according to the general procedure. The mixture was purified by column chromatography using EtOAc:Hex (2:8) to afford 31.1 mg of intermediate **21**(*17*) in 67.9% yield. The compound was hydrolysed to give 21.5 mg of carboxylic acid **22**(*17*) in 75.9% yield. The resulting carboxylic acid (21.5 mg, 0.075 mmol) was coupled with tryptamine (*6*) (12.0 mg, 0.075 mmol) according to the general procedure of amide-coupling to give, after column chromatography purification (EtOAc:Hex 7:3, R_f : 0.33), 18.5 mg of final product **24**(*17*) (yield: 57.7%). ^{1}H-NMR (600 MHz, ACETONE-D6) δ 10.02 (s, 0H), 9.14 (s, 1H), 8.24 (d, J = 2.4 Hz, 1H), 7.97 (t, J = 5.3 Hz, 1H), 7.72-7.67 (m, 2H), 7.47-7.45 (m, 3H), 7.39 (d, J = 7.9 Hz, 1H), 7.23 (d, J = 2.1 Hz, 1H), 7.11-7.09 (m, 1H), 7.04-7.01 (m, 1H), 6.96 (d, J = 2.1 Hz, 1H), 3.76 (dd, J = 13.6, 7.4 Hz, 2H), 3.11 (t, J = 7.4 Hz, 2H), 2.61 (s, 3H). LC-MS: *m/z* (ES+) 428.1 [M+H]+. HRMS calc. for $C_{25}H_{21}N_3O_4$ 427.45852, found 427.45194.

MTT cytotoxicity assay

Cell culture. The cell lines were grown at 37 °C in a 5% CO2 flux incubator and passaged when a confluence of 70-90% was reached, to either run the experiments or allow continued cell growth. No antibiotics were used in any part of this work. The growth curve of the cell lines was analysed to choose the optimal cell density. *Drug treatment*. Cells were seeded and once in logarithmic growth were incubated with compounds for 24 hours, and then removed. in the next step, the old medium was replaced by the fresh medium and incubated for further 48 hours. MTT/DMEM solution was added to the cells and incubated for 2 hours. Lastly, the MTT/DMEM solution was removed and DMSO was added to solubilize the formazan crystals. the absorbance was measured at 570 nm, subsequently, the cell viability (%) was calculated as (Absorbance value of formazan in treated cells-background/mean absorbance of negative control × 100).

Table S1. Structure of selected benzofuran-containing proximicin analogues belonging to Series 1 – 4 and antiproliferative activity (percentage viability) at a single concentration of 12 µg/mL against U-87 MG and WI-38 cells.

**Compound ID**	**Structures**	**% Viability U-87 MG** (12 µg/mL)	**% Viability WI-38** (12 µg/mL)
	**Series 1**		
8. NS-MHA5 **20**(*1*)	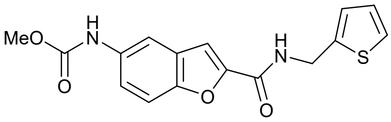	101.13	66.3
7. NS-MHA4 **20**(*2*)	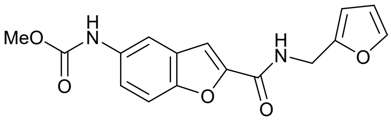	71.7	56.3
9. NS-MHA6 **20**(*3*)	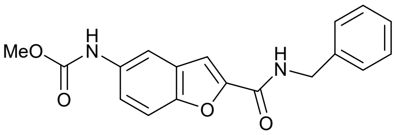	73.8	53.43
10. NS-MHA8 **20**(*4*)	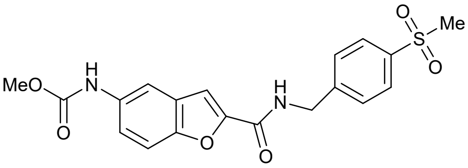	87.9	114.6
11. NS-MHA10 **20**(*5*)	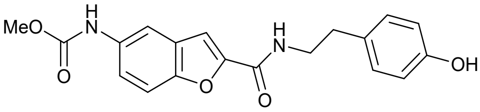	89.2	92.5
12. NS-MHA11 **20**(*6*)	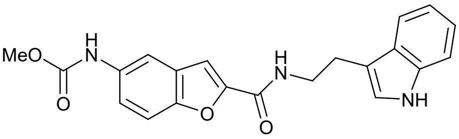	85.4	93.34
5. NS-MHA1 **20**(*7*)	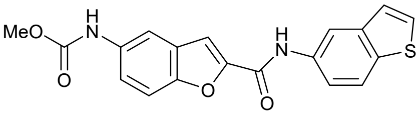	45.8	48.4
6. NS-MHA2 **20**(*8*)	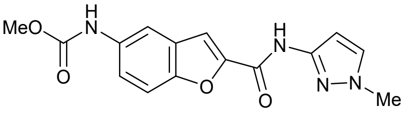	79.3	92.7
	**Series 2**		
1-NS-A9 **21**(*9*) 1	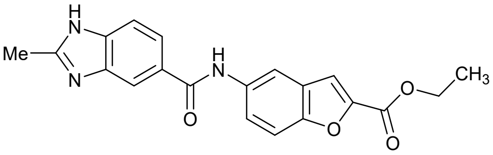	89.6	123.26

4. NS-25<br>**21**(*11*)<br>4	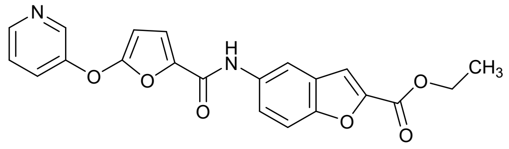	80.9	91.95
2. NS-A11<br>**21**(*18*)<br>2	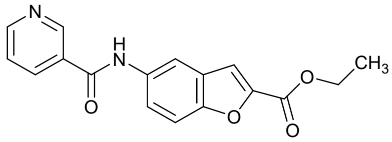	87.05	86.54
3. NS-A20<br>**21**(*19*)<br>3	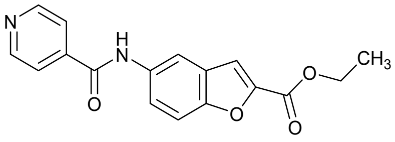	91.08	68.79
**Series 3**			
15. NS-AT5<br>**23**(*10*)	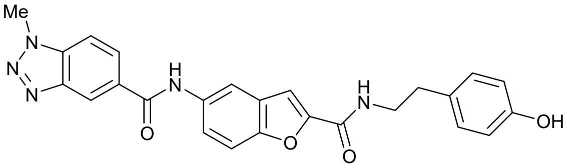	84.92	42.1
14. NS-AT4<br>**23**(*14*)	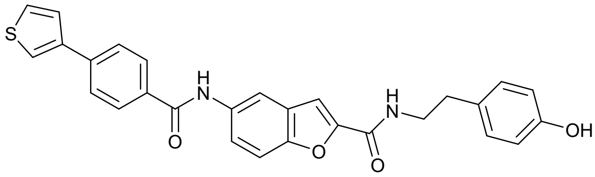	53.4	17.63
13. NS-AT1<br>**23**(*15*)	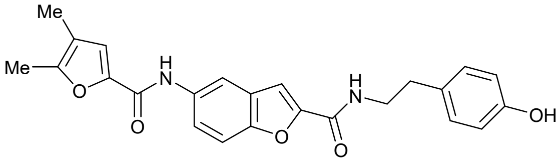	71.23	17.87
17. NS-AT10<br>**23**(*16*)	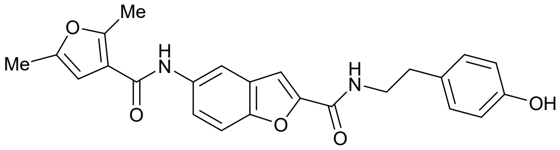	33.62	75.6
21. NS-AT23<br>**23**(*17*)	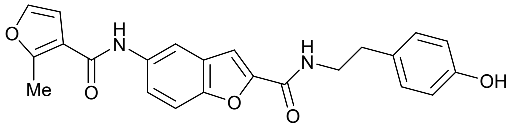	63.62	80.21
20. NS-AT22<br>**23**(*20*)	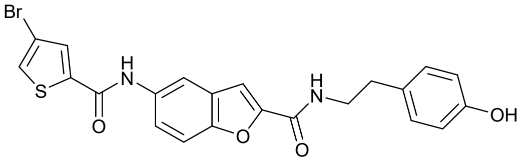	60.97	62.4
16. NS-AT7<br>**23**(*21*)	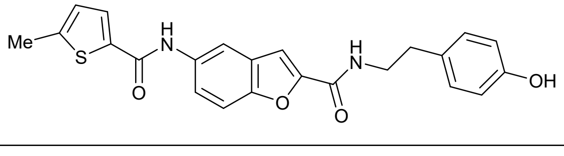	92.9	28.14

19. NS-AT17 **23**(*22*)	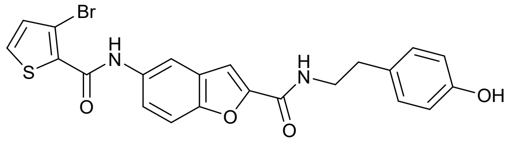	71.99	44.96
18. NS-AT15 **23**(*23*)	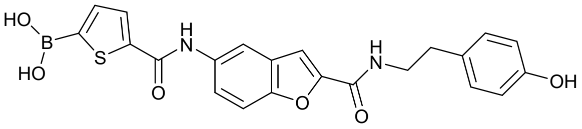	84.95	44.89
**Series 4**			
25. NS-ATP12 **24**(*12*)	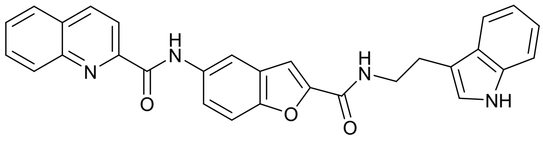	58.41	23.1
26. NS-ATP14 **24**(*13*)	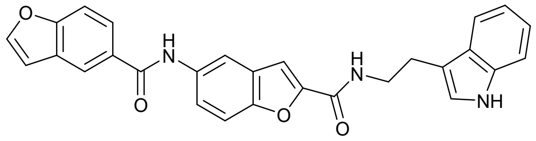	83.8	48.06
22. NS-ATP1 **24**(*15*)	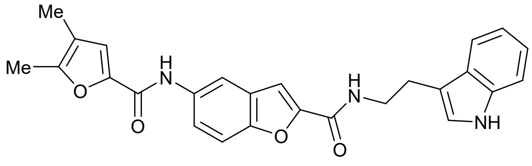	39.43	57.4
24. NS-ATP10 **24**(*16*)	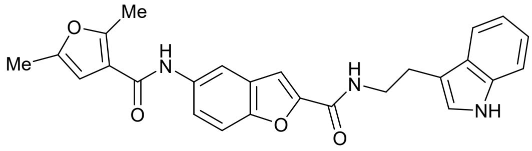	53.67	46.25
27. NS-ATP23 **24**(*17*)	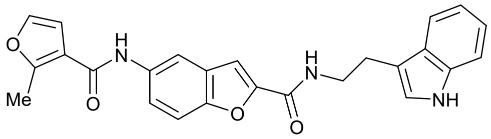	66.25	33.4
23. NS-ATP7 **24**(*21*)	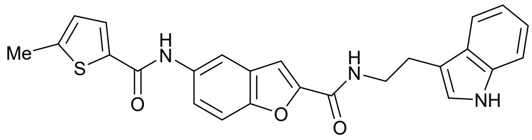	84.52	76.06
Temozolomide	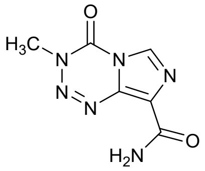	59.35	31.93

Figure S1. Growth curves for U87-MG and WI-38 cells using four different cell densities. In the graphs, the results of the growth curves experiments are displayed with (± SD values).

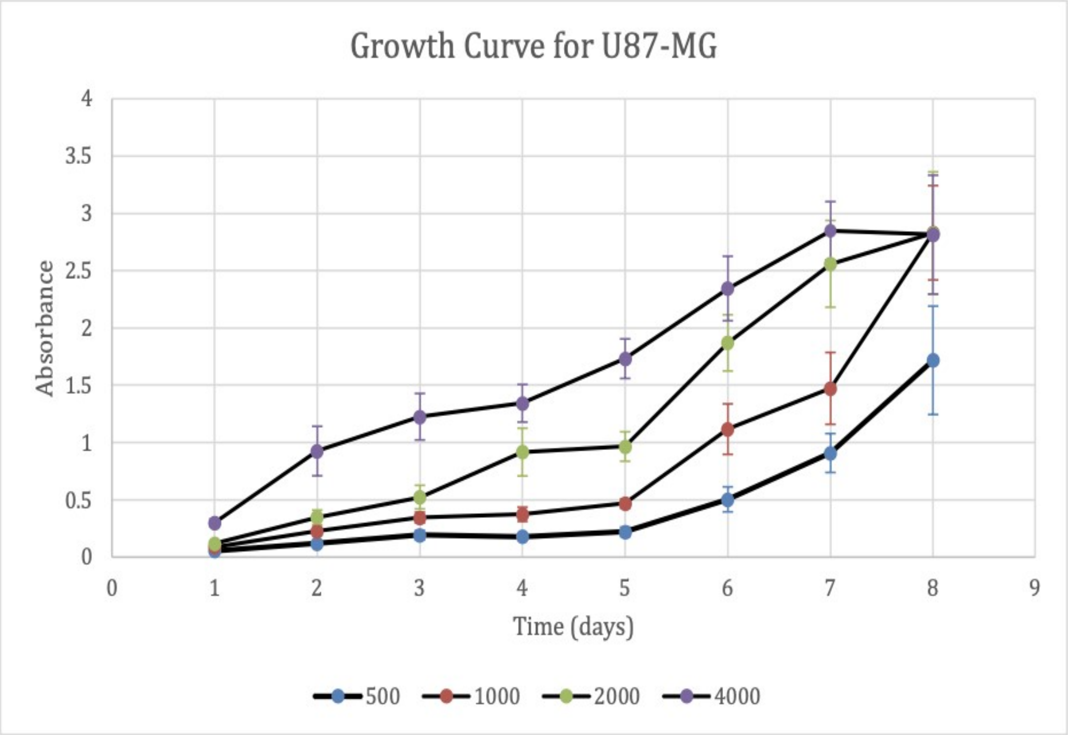

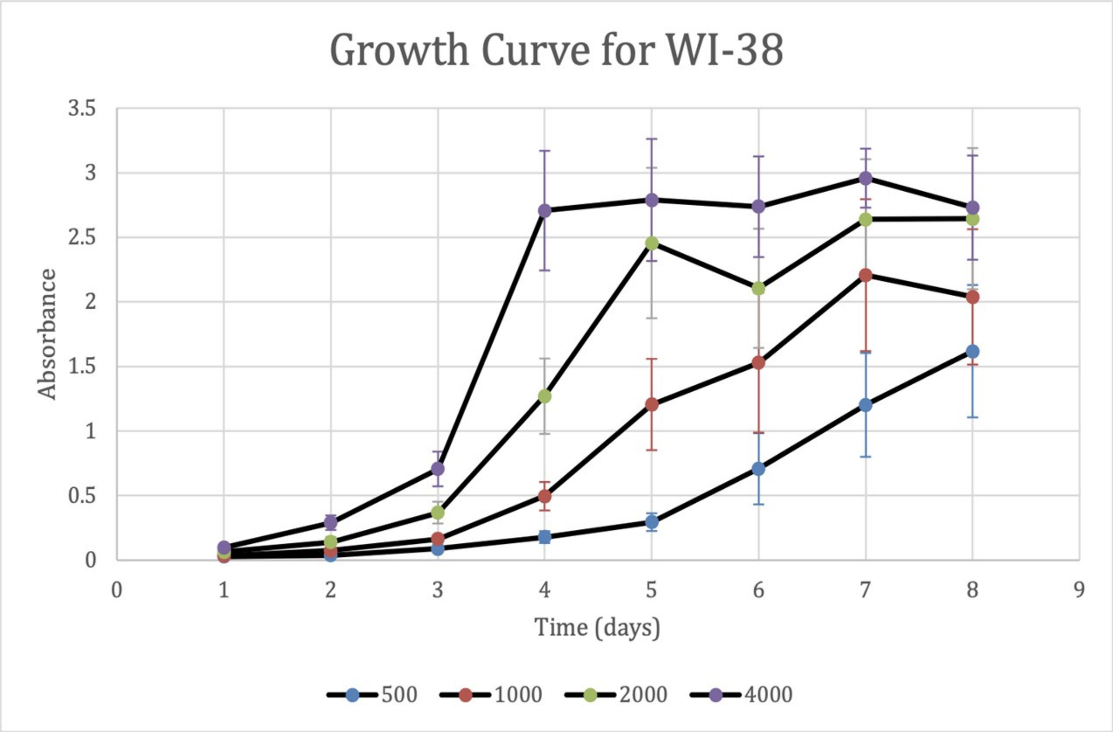

UHPLC-HRMS Data.

20(*6*) 12. NS-MHA11

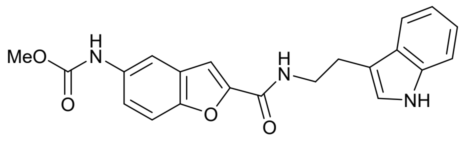

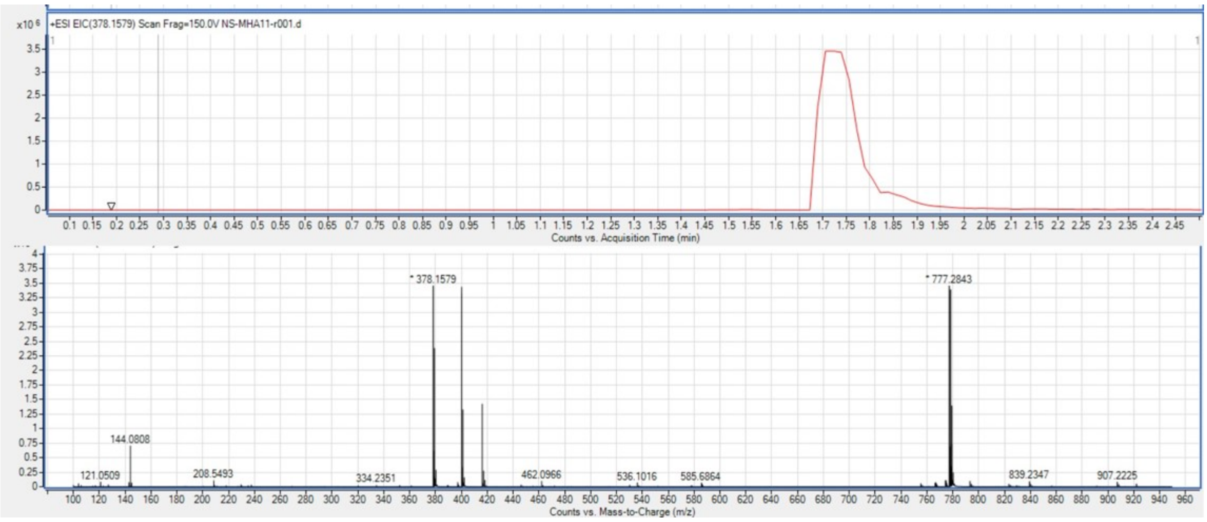

23(*22*) 19. NS-AT17

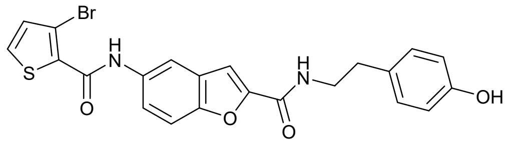

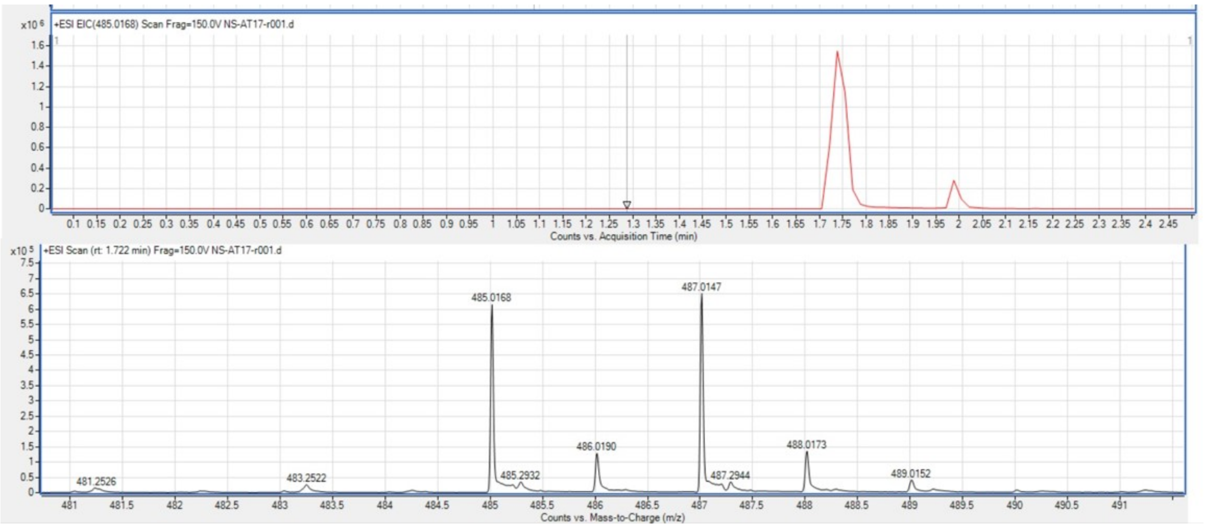

24(*15*) 22. NS-ATP1

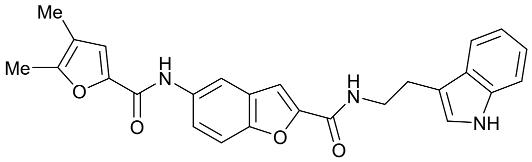

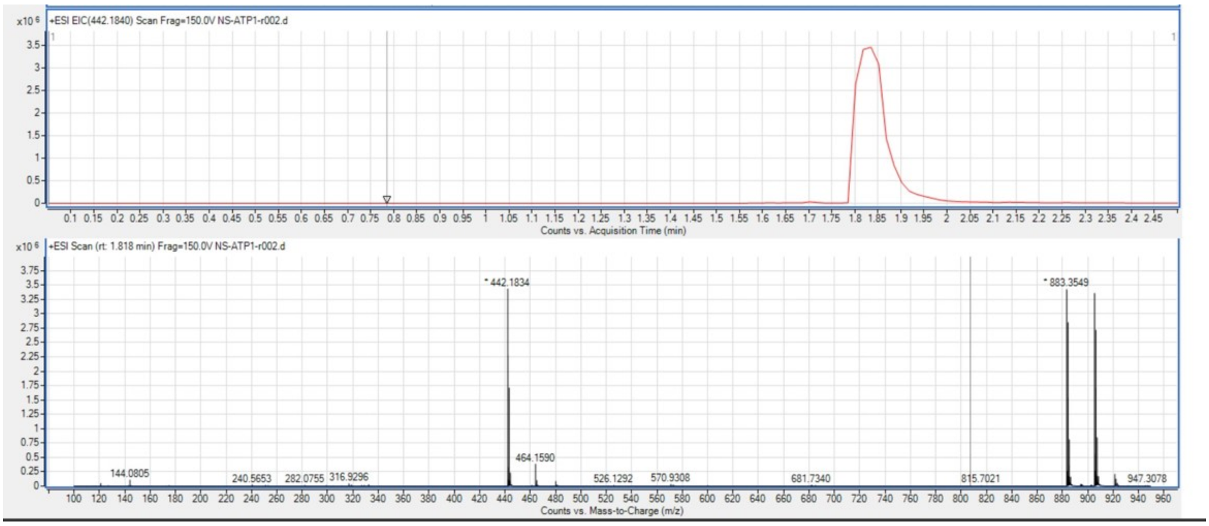

24(*12*) 25. NS-ATP12

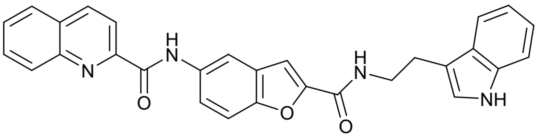

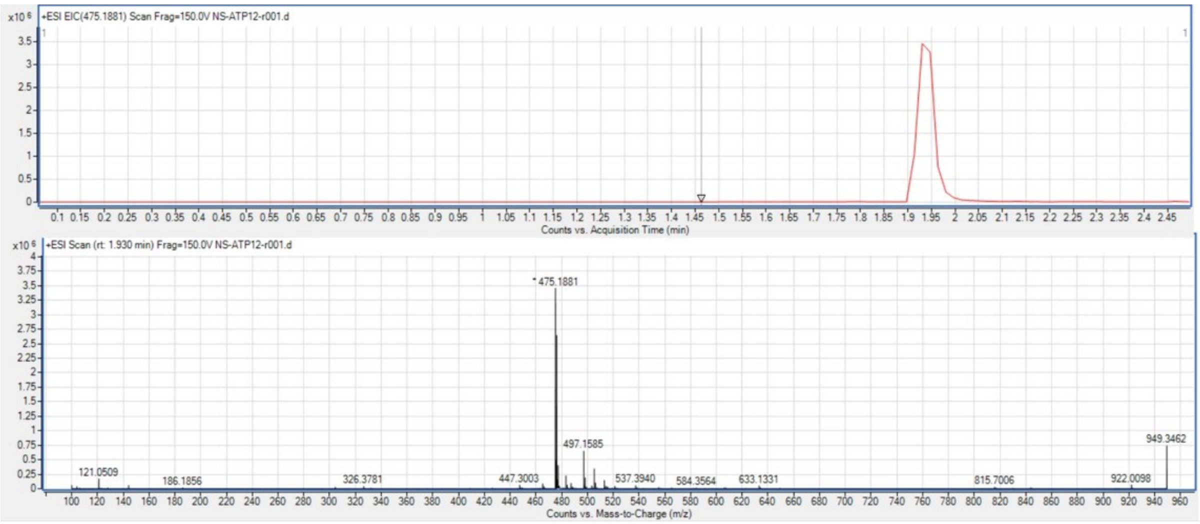

LC-MS data

21(*18*) 2. NS-A11 (MW = 310.09)

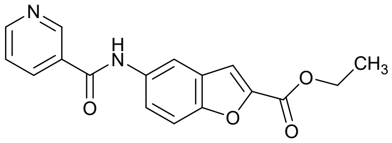

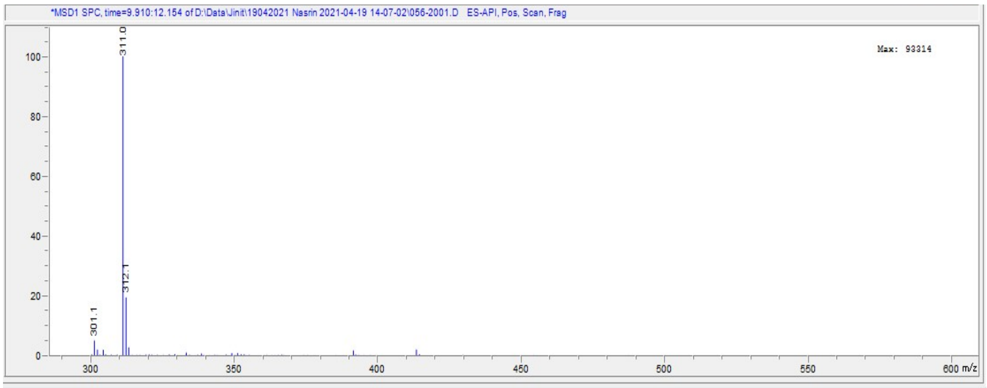

21(*19*) 3. NS-A20 (MW = 310.09)

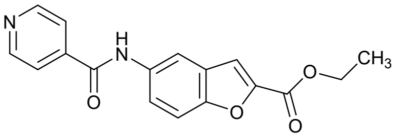

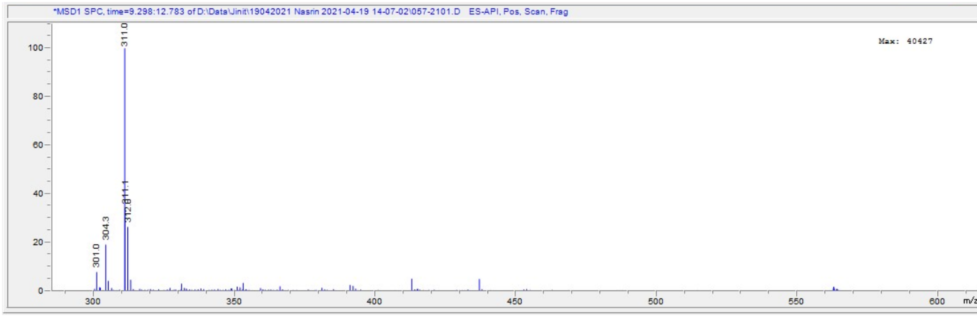

NMR-Data

Ethyl 5-Amino benzofuran-2-carboxylate ^{1}H-NMR

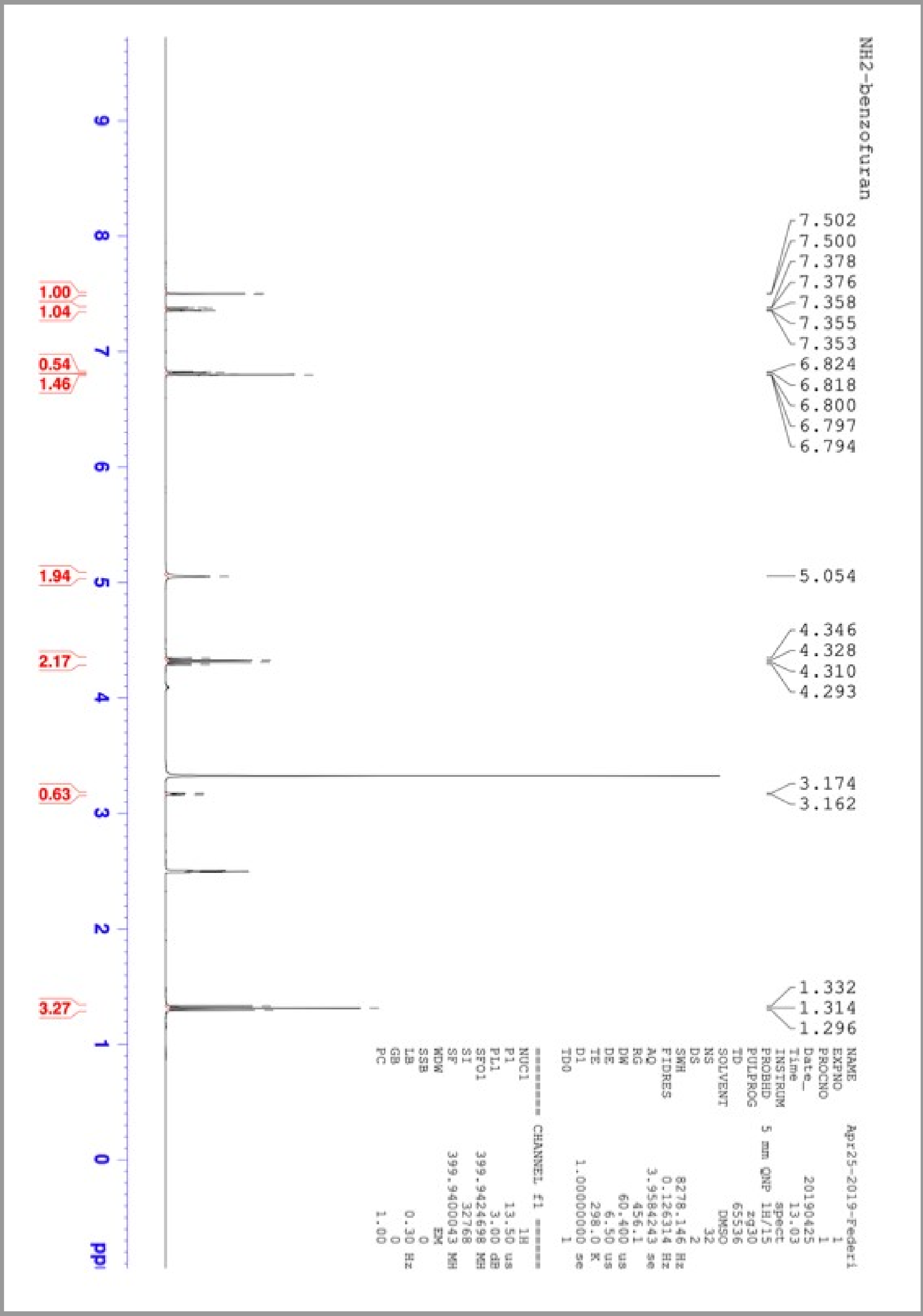

^{1}H NMR spectrum

21(*18*) 2. NS-A11

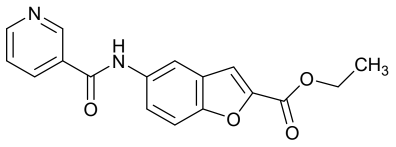

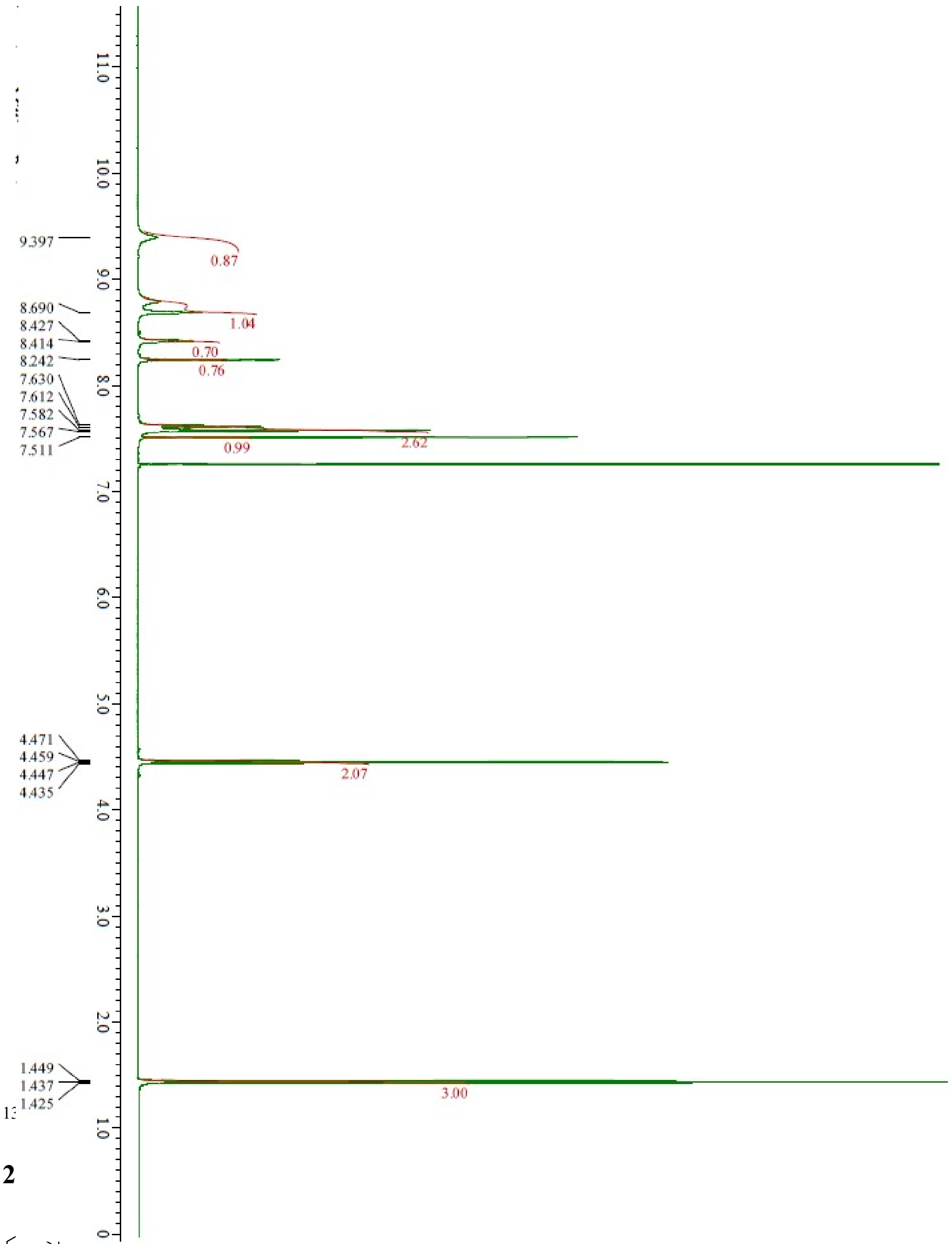

2

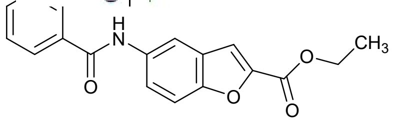

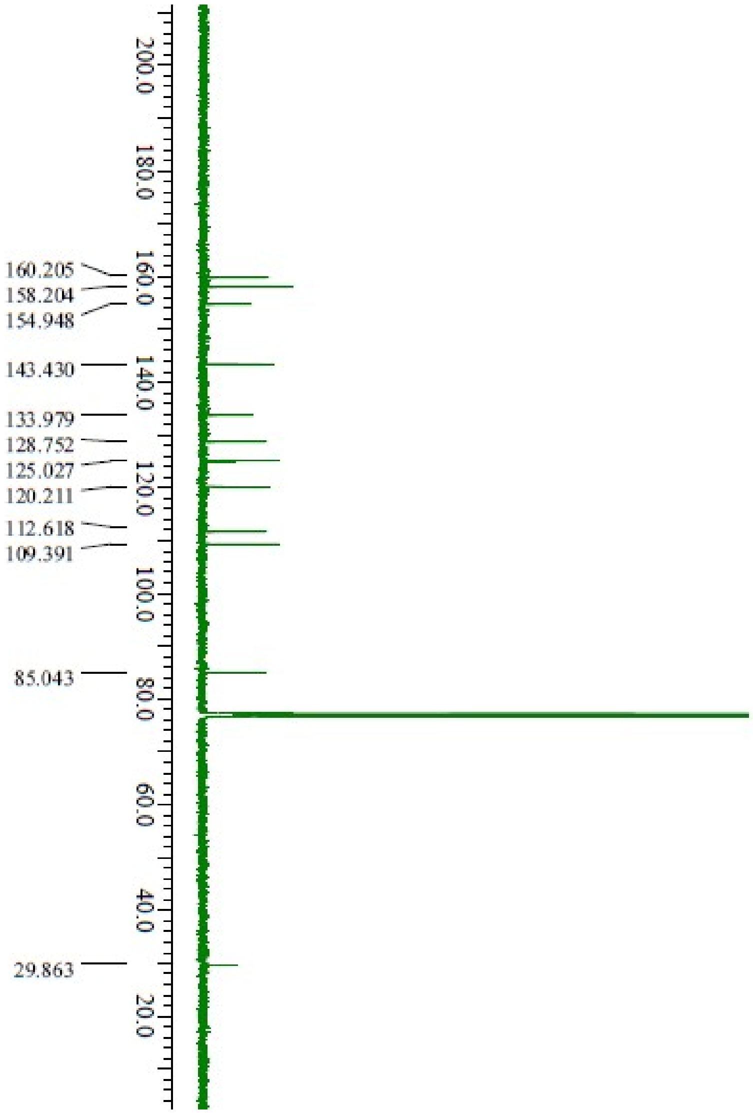
160.205
158.204
154.948
143.430
133.979
128.752
125.027
120.211
112.618
109.391
85.043
29.863
200.0
180.0
160.0
140.0
120.0
100.0
80.0
60.0
40.0
20.0

^{1}H NMR spectrum

21(*11*) 4. NS-A25

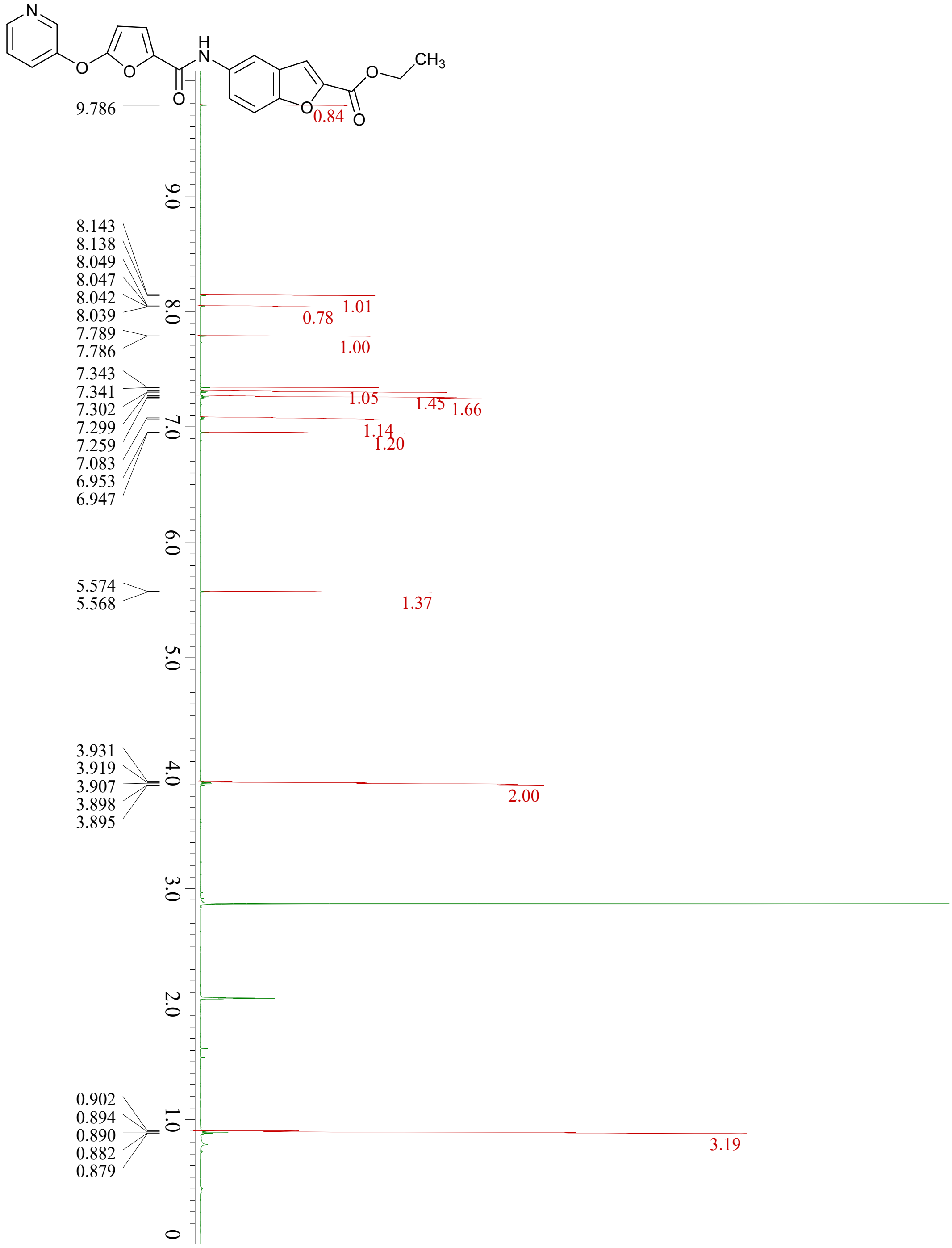

^{1}H NMR spectrum

20(*3*) 6. NS-MHA6

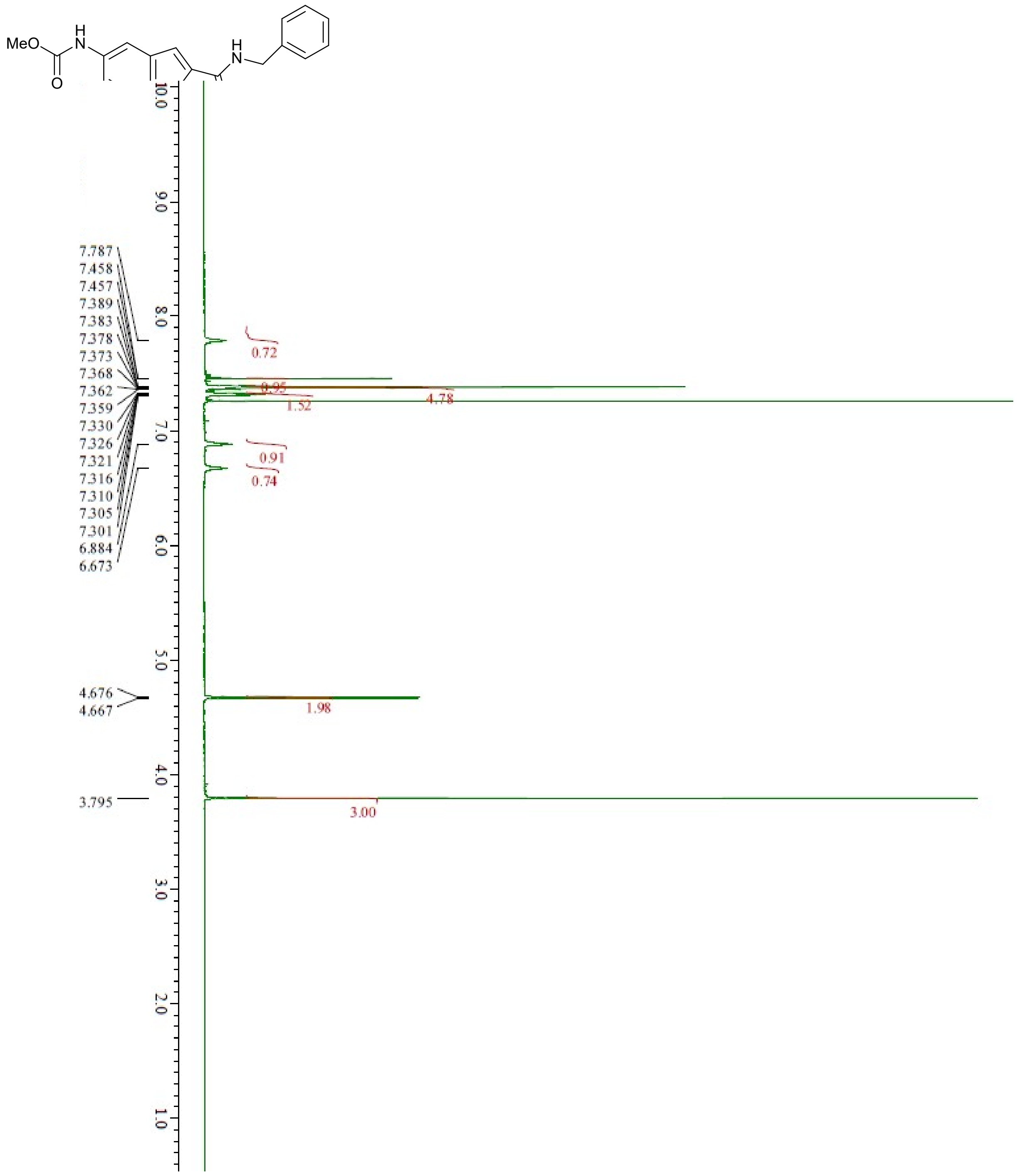

^{13}C NMR spectrum

20(*8*) 6. NS-MHA2

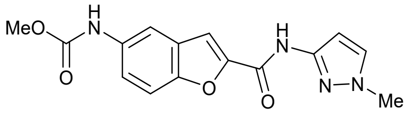

Chemical Formula: [illegible]

m/z: 314.10150 (100.[illegible]

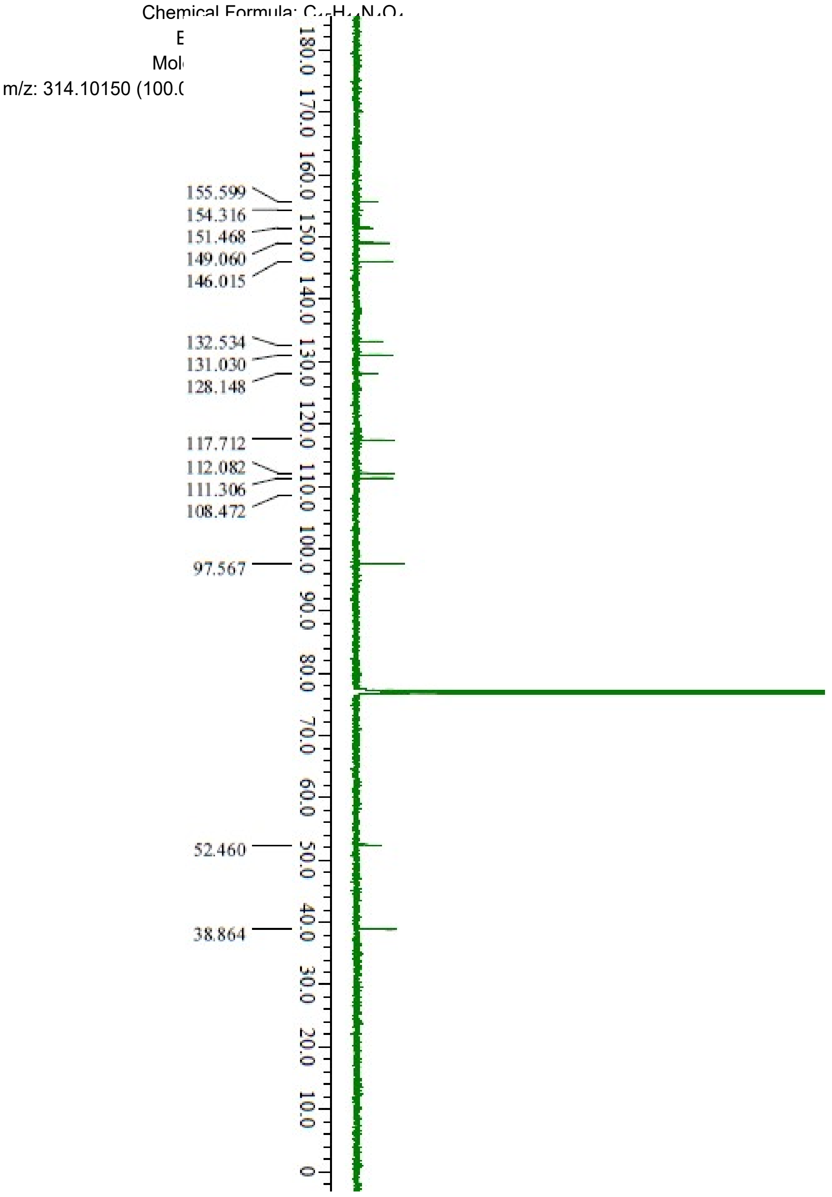

^{1}H NMR spectrum

23(*10*) 15. NS-AT5

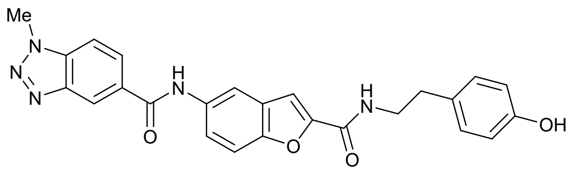

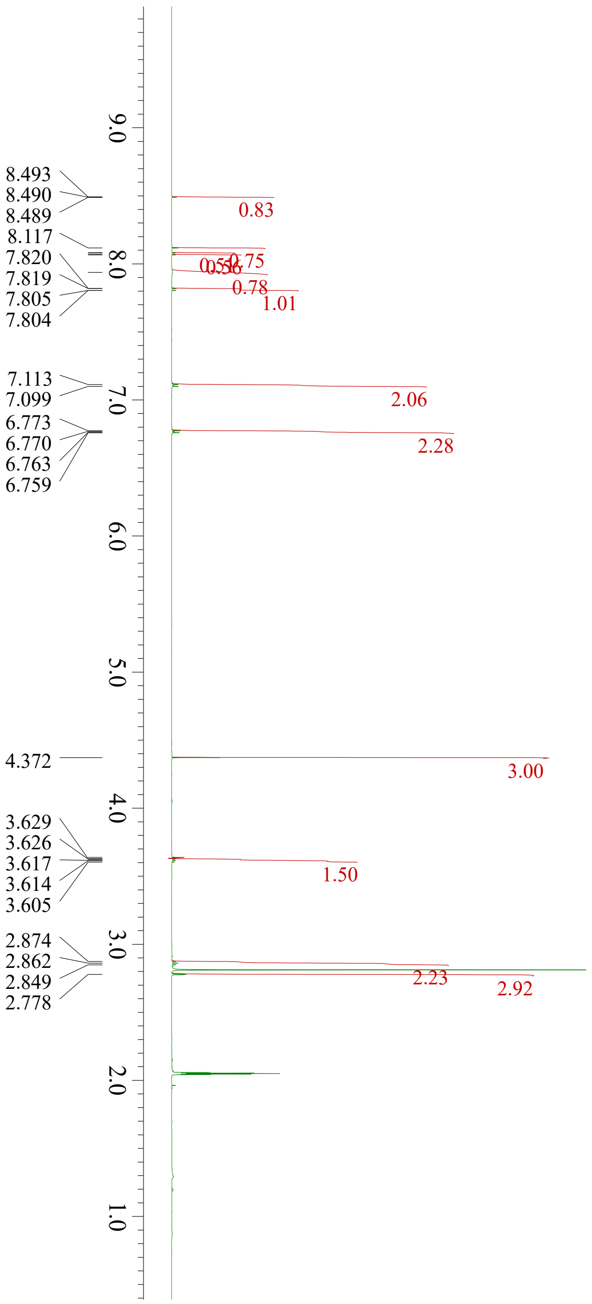

¹H NMR spectrum

23(*16*) 17. NS-AT10

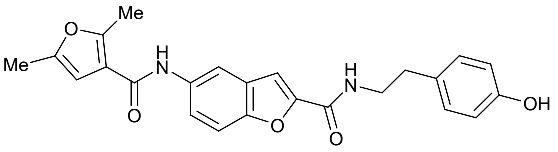

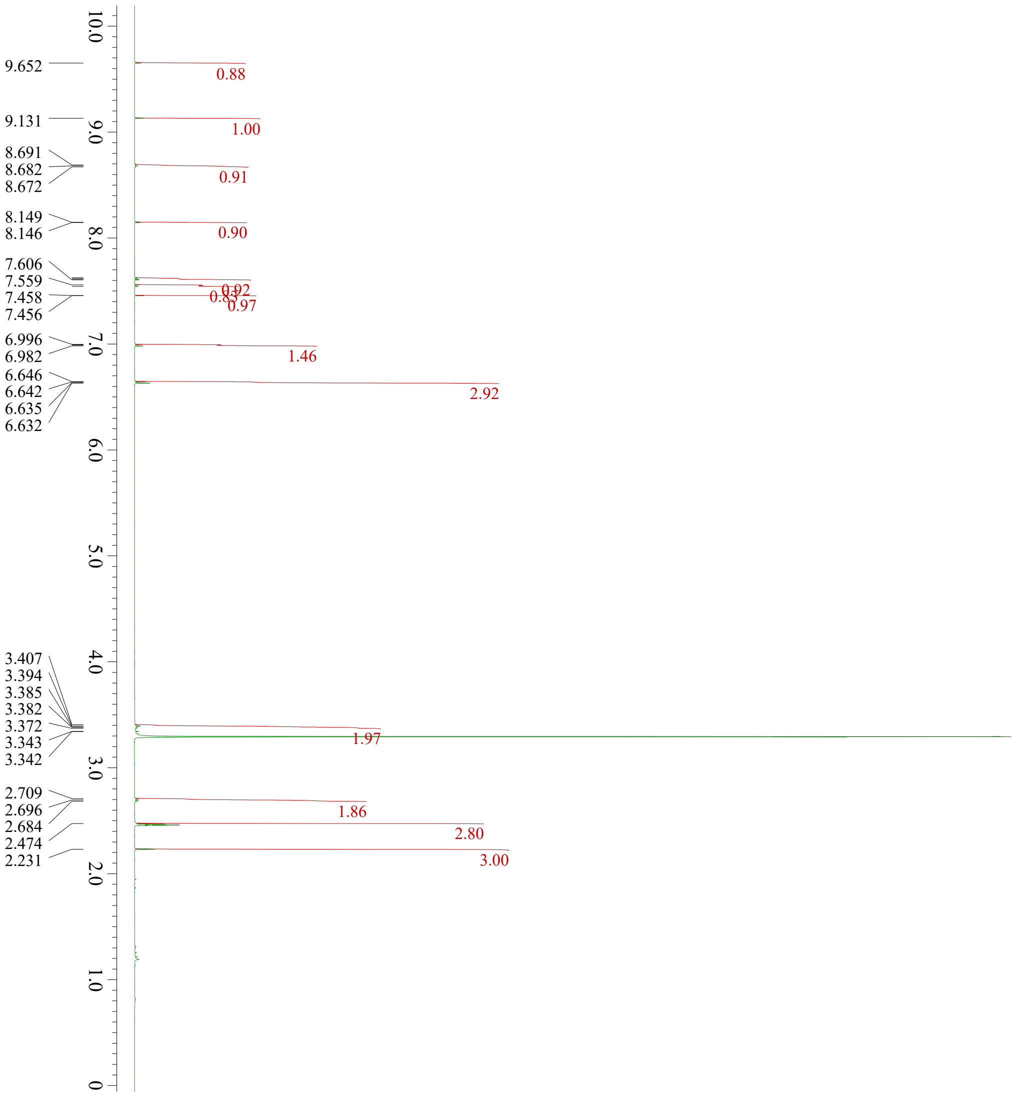

^{1}H NMR spectrum

23(*23*) 18. NS-AT15

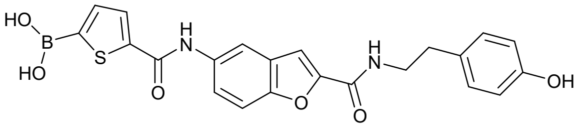

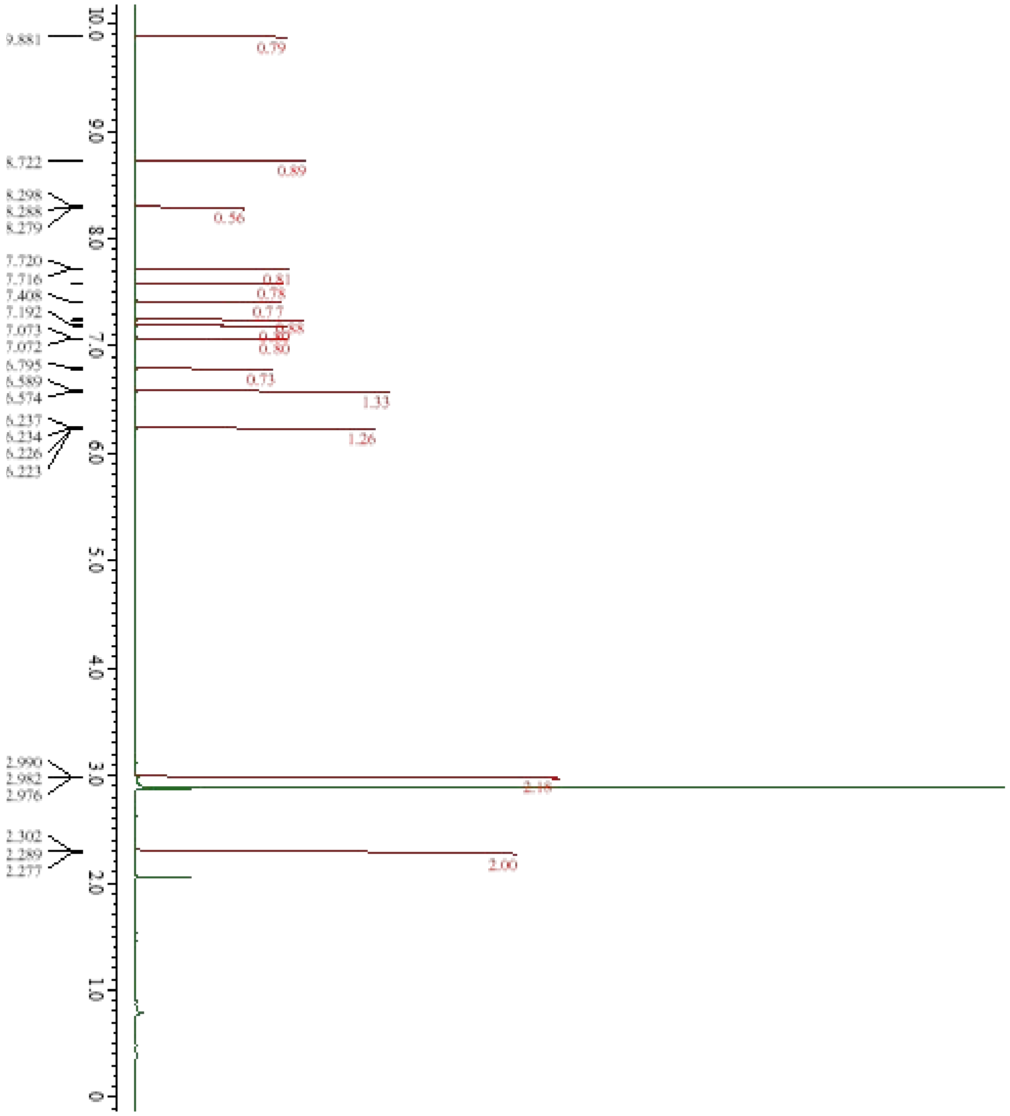

^{1}H NMR spectrum

24(*21*) 23. NS-ATP7

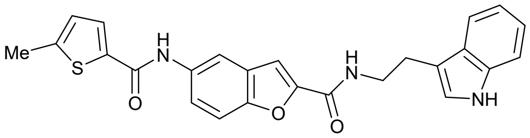

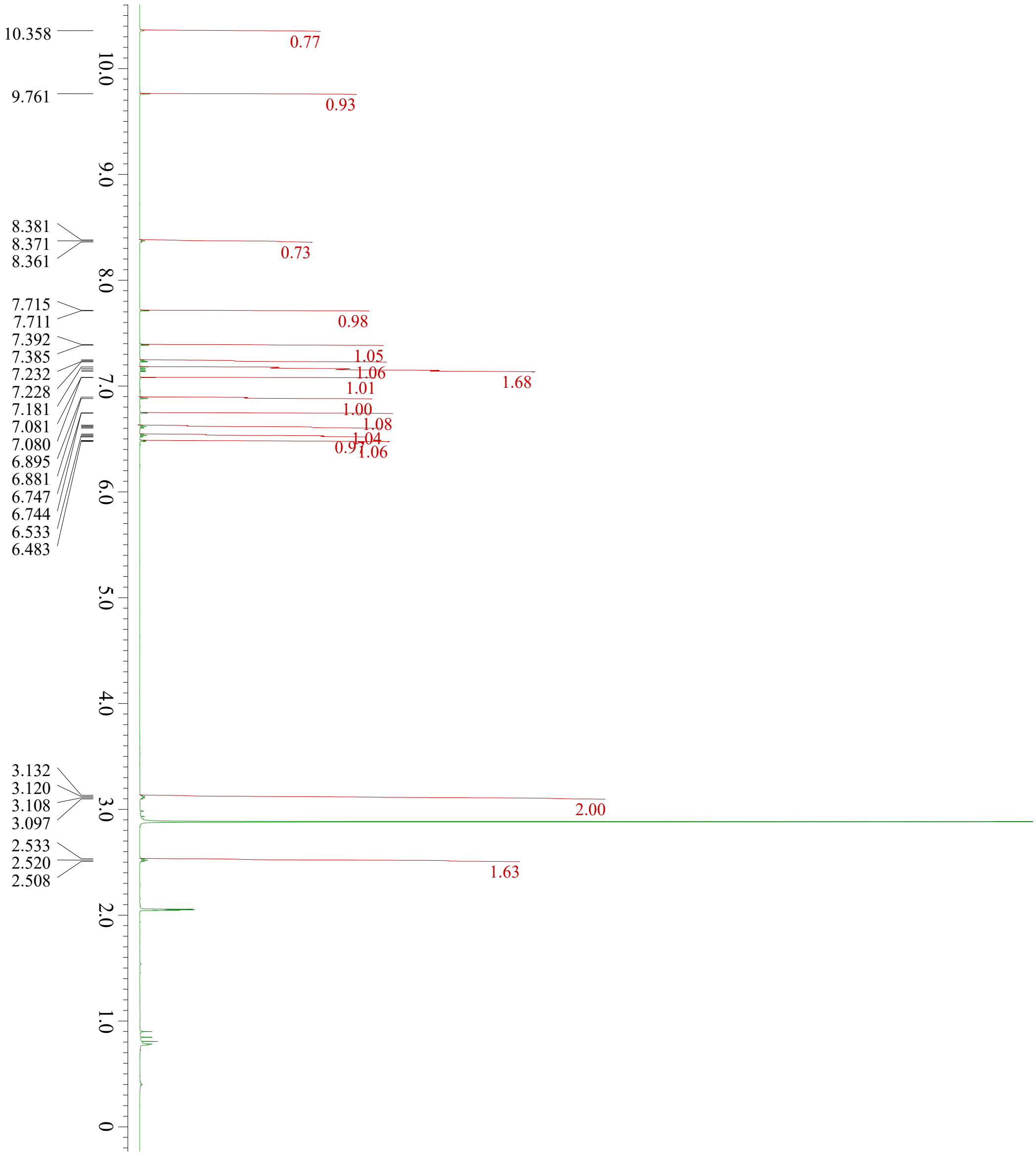

Supplement: RA-013-D3RA00107E-s001 [file RA-013-D3RA00107E-s001.pdf]
